# SPI1 activates mitochondrial unfolded response signaling to inhibit chondrocyte senescence and relieves osteoarthritis

**DOI:** 10.1038/s41413-025-00421-4

**Published:** 2025-04-14

**Authors:** Xiangyu Zu, Shenghong Chen, Zhengyuan Li, Lin Hao, Wenhan Fu, Hui Zhang, Zongsheng Yin, Yin Wang, Jun Wang

**Affiliations:** 1 Department of Oncology, The First Affiliated Hospital of Anhui Medical University, Anhui, China; 2 Department of Orthopaedics, The First Affiliated Hospital of Anhui Medical University, Anhui, China; 3 Anhui Province Key Laboratory of zoonoses, Anhui Medical University, Hefei, China; 4 Department of Wound Repair & Plastic and Aesthetic Surgery, the First Affiliated Hospital of Anhui Medical University, Anhui, China; 5 Anhui Public Health Clinical Center, Anhui, China

**Keywords:** Bone, Pathogenesis

## Abstract

Chondrocyte senescence is a critical pathological hallmark of osteoarthritis (OA). Aberrant mechanical stress is considered a pivotal determinant in chondrocyte aging; however, the precise underlying mechanism remains elusive. Our findings demonstrate that SPI1 plays a significant role in counteracting chondrocyte senescence and inhibiting OA progression. SPI1 binds to the PERK promoter, thereby promoting its transcriptional activity. Importantly, PERK, rather than GCN2, facilitates eIF2α phosphorylation, activating the mitochondrial unfolded protein response (UPR^mt^) and impeding chondrocyte senescence. Deficiency of SPI1 in mechanical overload-induced mice leads to diminished UPR^mt^ activation and accelerated OA progression. Intra-articular injection of adenovirus vectors overexpressing SPI1 and PERK effectively mitigates cartilage degeneration. In summary, our study elucidates the crucial regulatory role of SPI1 in the pathogenesis of chondrocyte senescence by activating UPR^mt^ signaling through PERK, which may present a novel therapeutic target for treating OA.

**Figure:**
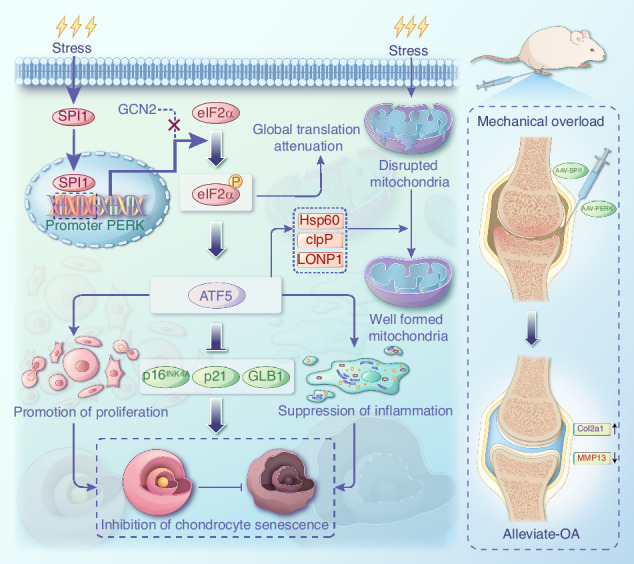
SPI1 alleviates the progression of OA by inhibiting mechanical stress-induced chondrocyte senescence through mitochondrial UPR signaling.

SPI1 alleviates the progression of OA by inhibiting mechanical stress-induced chondrocyte senescence through mitochondrial UPR signaling.

## Introduction

Osteoarthritis (OA) is a prevalent chronic degenerative joint disease characterized by a low cure rate and a progressive nature.^1^ The degeneration of articular cartilage (AC) represents a significant pathological feature of OA.^2,3^ Under normal physiological conditions, AC experiences increased metabolic and matrix synthesis activity in response to mechanical loading. However, abnormal mechanical stress can expedite AC degeneration by eliciting specific signals within the cartilage.^4^ Cartilage cell senescence denotes the irreversible cessation of cartilage cell growth, and the secretion of substantial amounts of inflammatory factors (senescence-associated secretory phenotype, SASP) also plays a pivotal role in AC degeneration.^5^ Nevertheless, the potential mechanisms linking abnormal mechanical stress, cartilage cell senescence, and OA remain unclear.

SPI1, also known as PU.1, is a member of the ETS (E-twenty-six) family of transcription factors and plays a pivotal role in the differentiation of various immune cells. SPI1 has been extensively studied for its involvement in immune cell-related tumors,^6^ and dysregulated SPI1 functions may trigger a DNA damage response, leading to fibroblast senescence. Furthermore, SPI1 modulates hematopoietic stem cell aging through the p38/MAPK14 signaling pathway and affects the DNA-binding capacity of these cells.^7^ Bioinformatics analysis suggests increased expression levels of SPI1 in synovial tissue, indicating a potential role in synovial inflammation associated with OA.^8^ Although it is implicated in regulating cell aging, there is currently insufficient evidence regarding its specific role in OA cartilage and chondrocyte aging.

Proteins within mitochondria play complex roles in mitochondrial function and are protected by the mitochondrial unfolded protein response (UPR^mt^).^9^ The UPR^mt^ is a mitochondrial-to-nuclear response that is activated when mitochondrial protein homeostasis is disrupted. The UPR^mt^ regulates protein folding, assembly, and degradation to maintain normal mitochondrial function by inducing mitochondrial protein quality control systems, including HSP60, LONP1, and ClpP.^10^ This mechanism has been shown to improve mitochondrial health during nematode development and extend the lifespan of nematodes, but studies in mammalian cells are still limited. Recent studies have shown that chromatin remodeling during mitochondrial dysfunction enhances UPR^mt^ activation in aged cells, promoting longevity.^11,12^ However, abnormal or prolonged UPR^mt^ activation can leave cells in a constant state of mitochondrial recovery, increasing the accumulation of deleterious mitochondrial genomes containing loss-of-function mutations or deletions (ΔmtDNAs) during development and aging. This accumulation may directly contribute to age-related deterioration.^13,14^ Therefore, precise regulation of the UPR^mt^ is crucial to avoid these potential negative effects.

The activation of the UPR^mt^ in mammals involves the phosphorylation of eukaryotic translation initiation factor 2 subunit 1 (eIF2α) and the expression of CHOP, ATF4, and ATF5.^15^ This process triggers the transcription of mitochondrial proteases such as HSP60, LONP1, and ClpP in response to the UPR^mt^.^16^ GCN2 and PERK are key kinases involved in eIF2α phosphorylation, and when these enzymes are activated by amino acid depletion or ER dysfunction, their activation significantly increases the level of phosphorylated eIF2α.^17^ The expression of PERK genes was found to be decreased in aged rat brains^18^ and mouse kidneys,^19^ and the PERK-mediated endoplasmic reticulum stress response (UPR^ER^) regulates the reversal of senescence; thus, PERK gene expression can be explored as a potential therapeutic option for treating neurodegeneration in glioblastoma.^20^ Additionally, loxenatide alleviates high glucose-induced pancreatic β-cell senescence by regulating the PERK/eIF2α pathway.^21^ GCN2 is a multidomain protein, and GCN2-null flies with a complete diet have a normal lifespan but equally short lifespans when any essential amino acid is removed from their diet.^22^ Recent studies have indicated that activating the GCN2-mediated integrated stress response (ISR) can promote the production of SASP components.^23^ In summary, the subtle relationship between cellular senescence and the UPR^mt^ is becoming clearer. However, the role of the UPR^mt^ in OA chondrocyte senescence remains unclear.

In this study, an abnormal mechanical stress-induced chondrocyte senescence model and SPI1 conditional knockout mice were established to investigate the relationships among SPI1 regulation, PERK expression changes, and UPR^mt^ activation during OA progression. We found that SPI1 can activate the UPR^mt^, enhance mitochondrial function via the UPR^mt^, inhibit chondrocyte senescence, and alleviate cartilage degeneration, thus ameliorating OA progression both in vitro and in vivo. In summary, our study revealed the significant protective role of SPI1-mediated activation of the PERK-UPR^mt^ signaling pathway in the progression of OA, providing potential diagnostic and therapeutic strategies for treating OA.

## Results

### SPI1 and PERK expression was significantly decreased in osteoarthritic articular cartilage

**Fig. 1:**
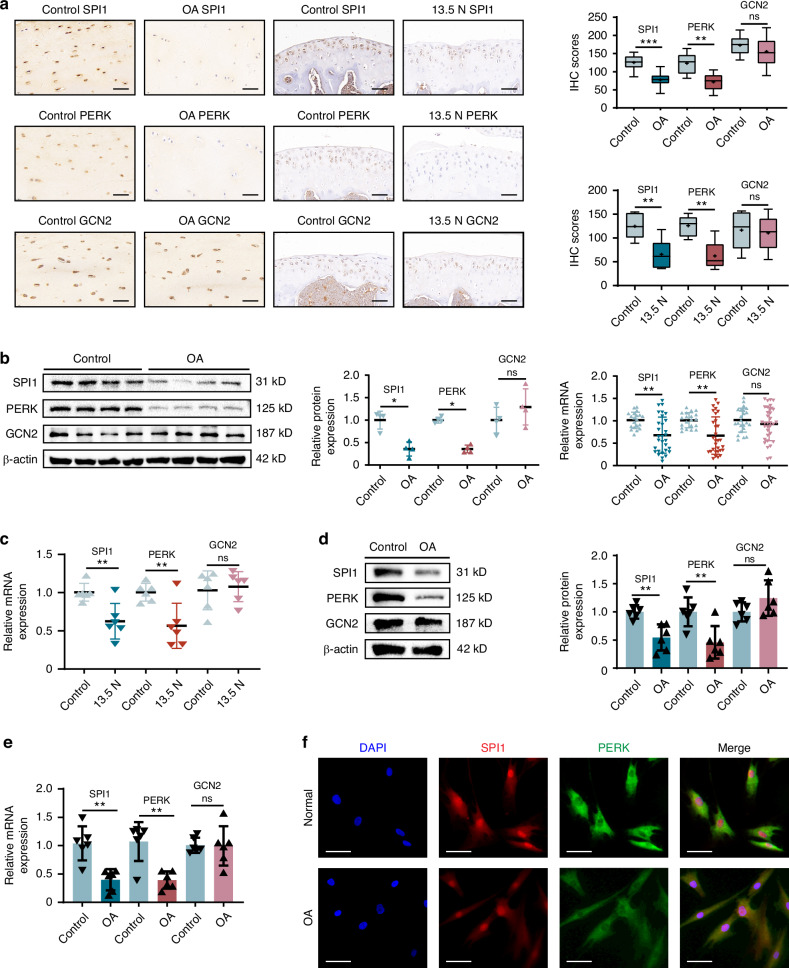
SPI1 and PERK expression was significantly decreased in osteoarthritic articular cartilage. **a** IHC of SPI1, PERK, and GCN2 expression in cartilage from OA patients (*n* = 10) and mechanical overload-induced mice (*n* = 6). Representative images and IHC scores are shown. Scale bars: 50 μm. **b** WB analysis of SPI1, PERK and GCN2 protein levels in cartilage from OA patients (*n* = 4). RT-qPCR was used to detect the mRNA expression of SPI1, PERK, and GCN2 in cartilage tissues of OA patients (*n* = 30) and control groups (*n* = 20). **c** RT-qPCR of SPI1, PERK and GCN2 mRNA expression in cartilage tissues of mechanical overload-induced model mice and control mice (*n* = 6 per group). **d** WB analysis of SPI1, PERK and GCN2 expression in chondrocytes of OA patients and control groups (*n* = 6 per group). **e** RT-qPCR of SPI1, PERK and GCN2 mRNA expression in chondrocytes of OA patients and control groups (*n* = 6 per group). **f** IF was used to observe the expression sites of SPI1 and PERK in OA chondrocytes and control groups (*n* = 4 per group). Scale bars: 50 μm. The experiments were independently repeated three times. Data are expressed as mean ± SD. *P < 0.05, **P < 0.01, ***P < 0.001 versus the control group. ns indicates not significant. *t*-test

The incidence of OA increases with age, and senescent cell clearance may attenuate OA development.^24^ Because SPI1 and the UPR^mt^ play important roles in regulating cellular senescence,^6,7,25^ we questioned whether SPI1 is involved in chondrocyte senescence in OA. First, immunohistochemistry (IHC) was performed on cartilage from OA patients, and the results revealed that the expression of SPI1 and PERK in OA patient cartilage was lower than that in the control group, whereas GCN2 expression did not significantly change (Fig. 1a). Next, we established an in vivo mechanical loading mouse model. Safranin O-Fast Green staining revealed significant cartilage degeneration in the mice subjected to 13.5 N mechanical overloading compared with the control group, whereas no noticeable changes were observed under lower mechanical loads of 4 N or 9 N (Fig. S1a). We performed IHC staining on the joints of the successfully established mechanical overload mouse model. The results revealed that the expression of SPI1 and PERK in mechanical overload-induced model mice was lower than that in control mice, whereas GCN2 expression did not significantly change. We also observed that SPI1-positive staining was localized mainly in the nucleus and that PERK-positive staining was localized mainly in the cytoplasm (Fig. 1a). This phenomenon was consistent in aged mice (20-month-old), in which SPI1 and PERK expression was significantly decreased compared with young mice (3-month-old), while GCN2 showed no significant change (Fig. S1b-c). The protein and mRNA levels of SPI1 and PERK were decreased in human OA cartilage tissue, whereas those of GCN2 were not significantly changed (Fig. 1b). The mRNA levels of SPI1 and PERK were decreased in mechanical overload-induced mouse cartilage tissue, which is consistent with previous results, whereas GCN2 also showed no significant changes (Fig. 1c). We subsequently cultured chondrocytes extracted from human cartilage tissue and observed that the protein and mRNA levels of SPI1 and PERK were significantly lower in OA chondrocytes than in normal control chondrocytes, while GCN2 also showed no significant changes (Fig. 1d, e). When chondrocytes were subjected to immunofluorescence (IF), SPI1 and PERK were expressed in both the nucleus and the cytoplasm; SPI1 was predominantly localized in the nucleus, whereas PERK was primarily localized in the cytoplasm. Immunofluorescence further revealed that the localization levels of SPI1 and PERK in both the cytoplasm and nucleus were lower in OA chondrocytes than in control cells (Fig. 1f). These cues establish a direct association between SPI1 and PERK reduction and OA.

### SPI1 and PERK are involved in mechanical stress-induced chondrocyte senescence

**Fig. 2:**
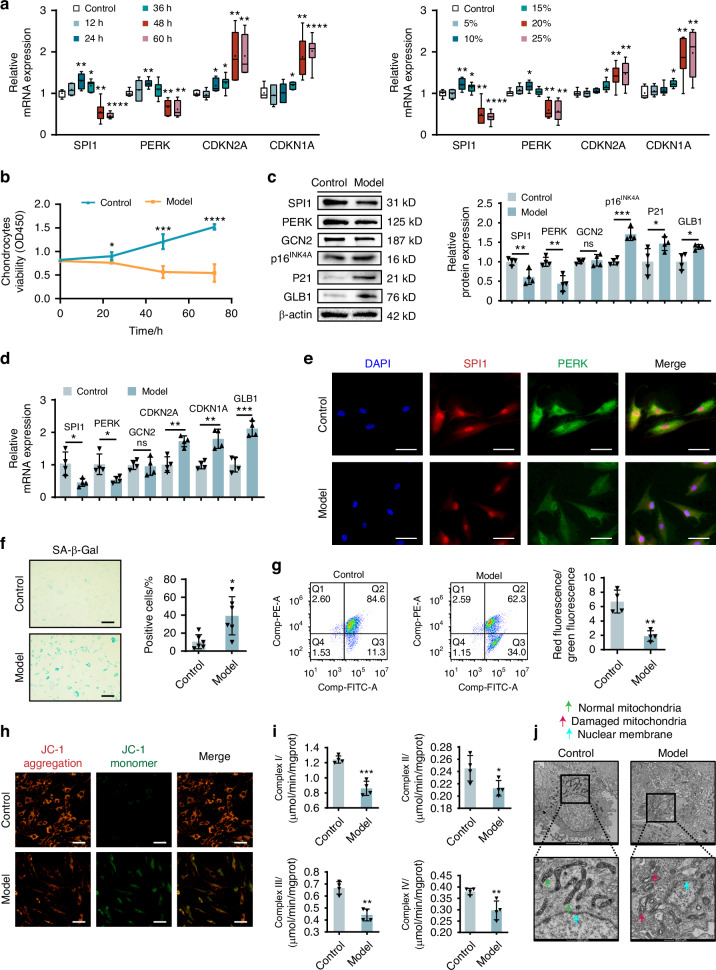
SPI1 and PERK are involved in mechanical stress-induced chondrocyte senescence. **a** RT-qPCR of SPI1, PERK, CDKN2A, CDKN1A mRNA expression in the chondrocytes under mechanical stress time and stress gradient (*n* = 6 per group). **b** The CCK-8 was used to assess the proliferative activity of chondrocytes under 20% mechanical stress. **c**, **d** WB and RT-qPCR analyses were performed to detect the expression of SPI1, PERK, GCN2, p16^INK4A^, p21, and GLB1 in chondrocytes subjected to 48 h of 20% mechanical stress (model groups). **e** Representative IF images of SPI1 and PERK expression in model groups and control groups. Scale bars: 50 μm. **f** The model groups and control groups were analyzed by β-galactosidase staining (*n* = 6 per group). Scale bars: 50 μm. **g**, **h** Flow cytometry (JC-1) and microscopy were used to observe the mitochondrial membrane potential of model groups and control groups. Scale bars: 50 μm. **i** Mitochondrial respiratory chain complexes I-IV were determined in model groups and control groups. **j** TEM was used to observe the morphology of mitochondria in model groups and control groups. Scale bars: 2 μm and 500 nm. The cell sample size is *n* = 4. Experiments were independently repeated three times. Data are expressed as mean ± SD. *P < 0.05, **P < 0.01, ***P < 0.001, ****P < 0.000 1 versus the control group. ns indicates not significant. *t*-test

Abnormal mechanical stress accelerates articular cartilage degeneration by activating specific signals in chondrocytes. Mechanical overload also accelerates the aging process of cultured chondrocytes and mouse articular cartilage.^4,26^ We isolated and cultured chondrocytes from human OA cartilage to investigate the correlation between SPI1 and PERK and mechanical stress-induced chondrocyte senescence. Subsequently, abnormal mechanical stress was applied to induce chondrocyte senescence. First, we explored the stress magnitude gradient and time gradient of mechanical stress in chondrocytes. We found that chondrocyte senescence was most obvious at 48 h and 20% mechanical stress. SPI1 and PERK tended to first increase but then decrease, and SPI1 and PERK decreased most significantly at 48 h and 20% mechanical stress (Fig. 2a). We unexpectedly found that under mechanical stress, the levels of the UPR^mt^-related components HSP60, LONP1 and ClpP in chondrocytes tended to initially increase but then decrease over time with increasing mechanical stress, with the most pronounced changes observed at 48 h and 20% stress, while GCN2 also showed no significant changes. (Fig. S2a). Therefore, in all subsequent experiments, we used 20% mechanical stress for 48 h as the experimental condition. We found that with persistent abnormal stress, there was a gradual decrease in chondrocyte viability, with a significant reduction occurring approximately 48 h after pressure application (Fig. 2b). The protein and mRNA levels of SPI1 and PERK were significantly lower in the experimental groups than in the corresponding control groups. Additionally, increased expression of p16^INK4A^ (CDKN2A), p21 (CDKN1A), and GLB1 suggested successful induction of chondrocyte senescence due to mechanical stress. The expression level of GCN2 did not significantly change (Fig. 2c, d). IF staining revealed that, compared with that in the control group, the fluorescence intensity of SPI1 and PERK was lower in senescent cells. Notably, the nuclear fluorescence intensity of SPI1 was significantly decreased, especially in senescent cells (Fig. 2e). The proportion of β-galactosidase-positive cells significantly increased in the model group (Fig. 2f), and senescent chondrocytes expressed significantly higher levels of IL-6, IL-8, and TNF-α than did control cells, which are typical markers of cell aging (Fig. S2b). The flow cytometry results revealed a notable decrease in the mitochondrial membrane potential (Fig. 2g, h) in the senescent chondrocytes compared with the control chondrocytes. Furthermore, abnormal mechanical stress significantly inhibited mitochondrial complex I-IV activity, as shown by respiratory chain complexes I-IV within these cells (Fig. 2i). Transmission electron microscopy revealed a uniform distribution with intact double-membrane and crista structures within normal mitochondria; however, mitochondria within senescent cells exhibited irregular morphology along with disrupted or lost double-membrane and cristome structures (Fig. 2j). These findings suggest that abnormal mechanical stress can induce chondrocyte senescence through mechanisms potentially related to aberrant expression patterns of SPI1 and PERK.

**Fig. 3:**
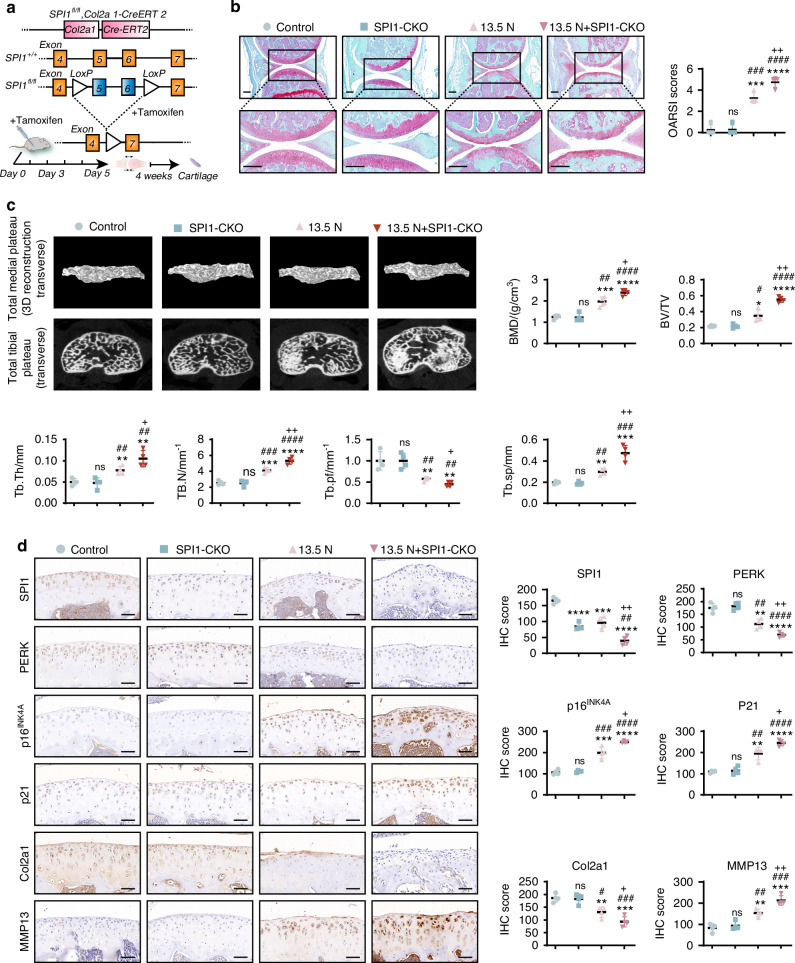
SPI1 and PERK are involved in mechanical stress-induced chondrocyte senescence. **a** Genome-editing strategy for generating inducible SPI1 knockout alleles. Col2a1-Cre-ERT2 mice carry the tamoxifen-responsive Cre recombinase transgene downstream of the Col2a1 promoter. SPI1^fl/fl^ mice contain two loxP sites flanking SPI1 exons 5 and 6. Intraperitoneal injection of tamoxifen in the adult mouse induces the nuclear translocation of the ubiquitously expressed Cre–ERT2 fusion protein, resulting in the excision of the genomic fragment located between the loxP sites to generate SPI1 null alleles. **b** Safranin O/fast green staining was used to assess cartilage destruction and OARSI scores in the articular cartilage of SPI1-CKO mice. Scale bar: 200 μm. **c** Analysis of BMD and knee joint bone histomorphometric parameters using micro-CT data in SPI1 CKO mice. Original magnification ×50. **d** IHC of SPI1, PERK, p16^INK4A^, p21, Col2a1 and MMP13 expression in cartilage tissues of SPI1-CKO mice. Scale bar: 50 μm. The sample size is *n* = 4. Experiments were independently repeated three times. The data were expressed as mean ± SD. *P < 0.05, **P < 0.01, ***P < 0.001, ****P < 0.000 1 versus the Control group. ^#^*P* < 0.05, ^##^*P* < 0.01, ^###^*P* < 0.001, ^####^*P* < 0.000 1 versus the SPI1-CKO group. ^+^*P* < 0.05, ^++^*P* < 0.01 versus the 13.5 N group. ns indicates not significant. One-way ANOVA

To further investigate the role of SPI1 in mechanical stress-induced chondrocyte senescence, we generated SPI1 conditional knockout mice (SPI1^f/f^Col2a1-CreERT2) (Fig. 3a), with SPI1^f/f^ wild-type littermates used as controls. The results of Safranin O/Fast Green staining indicated that, compared with wild-type mice, SPI1 knockout mice without mechanical stress did not exhibit more severe OA or age-related phenotypes. In contrast, soft tissue degeneration and age-related phenotypes were more pronounced in the 13.5 N group and the 13.5 N + SPI1-CKO group than in the control group, suggesting that the mechanical stress model successfully induced OA. Under mechanical stress conditions, SPI1 knockout exacerbated joint damage. Furthermore, compared with the SPI1-CKO group, the 13.5 N + SPI1-CKO group presented significantly more severe OA and age-related phenotypes (Fig. 3b). Micro-CT analysis revealed a significant increase in bone sclerosis in SPI1 conditional knockout mice following mechanical overload. However, compared with wild-type mice, SPI1 knockout mice without mechanical overload did not exhibit significant changes in bone sclerosis (Fig. 3c). Additionally, IHC analysis revealed a significant decrease in the expression levels of SPI1, PERK and Col2a1 in the cartilage of SPI1 knockout mechanical overload-induced mice, along with a notable increase in the expression levels of p16^INK4A^, p21 and MMP13. Moreover, compared with those in wild-type mice, the expression levels of PERK, p16^INK4A^, p21, Col2a1, and MMP13 in SPI1 knockout mice without mechanical overload did not significantly change (Fig. 3d). These findings suggest a close relationship between SPI1 and the regulation of mechanical stress-induced chondrocyte senescence.

### SPI1 inhibits the mechanical stress-induced senescence of chondrocytes

**Fig. 4:**
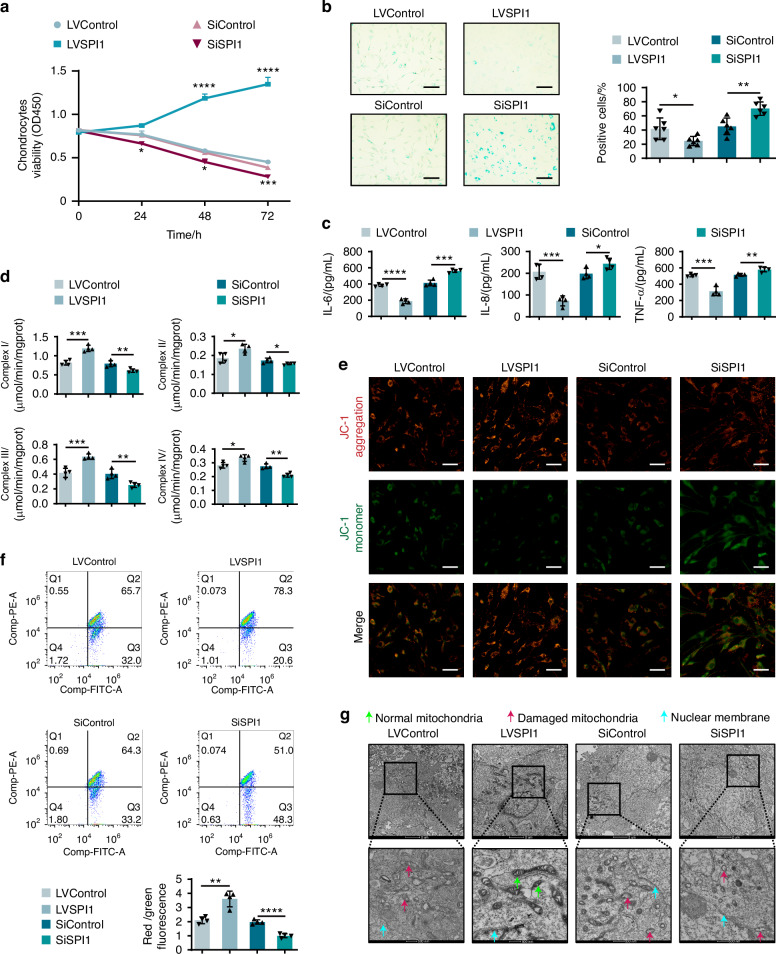
SPI1 inhibits the mechanical stress-induced senescence of chondrocytes. **a** CCK-8 was used to assess the proliferative activity of chondrocytes in model groups with SPI1 overexpression or inhibition. **b** β-Galactosidase staining was used to assess the chondrocytes in model groups with SPI1 overexpression or inhibition (*n* = 6 per group). Scale bars: 50 μm. **c** IL-6, IL-8, and TNF-α levels in the culture supernatant of chondrocytes with SPI1 overexpression or inhibition in model groups were measured by ELISA. **d** Mitochondrial respiratory chain complexes I-IV were assessed in chondrocytes with SPI1 overexpression or inhibition in model groups. **e**, **f** Mitochondrial membrane potential in chondrocytes with SPI1 overexpression or inhibition in model groups was measured by flow cytometry and observed by microscopy. Scale bars: 50 μm. **g** The morphology of mitochondria was observed by TEM in chondrocytes with SPI1 overexpression or inhibition in model groups. Scale bars: 2 μm and 500 nm. The cell sample size is *n* = 4. Experiments were independently repeated three times. The data were expressed as mean ± SD. *P < 0.05, **P < 0.01, ***P < 0.001, ****P < 0.000 1 versus the Control group. *t*-test

To further investigate the role of SPI1 in abnormal mechanical stress-induced chondrocyte senescence, SPI1 was overexpressed (LVSPI1), and SPI1 was knocked down (SiSPI1) in a senescent chondrocyte model. Control: senescent chondrocytes were transfected with LVControl or SiControl. We found that LVSPI1 reversed the inhibitory effect of abnormal stress on chondrocyte proliferation and suppressed cell aging, whereas SiSPI1 transfection further reduced the survival ability of chondrocytes and increased aging (Fig. 4a, b). Additionally, treatment with LVSPI1 inhibited the expression of IL-6, IL-8, and TNF-α in chondrocytes under abnormal mechanical stress (Fig. 4c). Notably, treatment with LVSPI1 can augment the activity of respiratory chain complexes I-IV and reverse the decline in the mitochondrial membrane potential induced by abnormal mechanical stress. In contrast, SiSPI1 transfection decreased the activity of respiratory chain complexes I-IV and further reduced the mitochondrial membrane potential compared with that in senescent chondrocytes (Fig. 4d-f). Microscopic observation of the mitochondrial membrane potential was consistent with our previous observations, where LVSPI1 restored the mitochondrial membrane potential, and SiSPI1-treated chondrocytes presented a reduction in the mitochondrial membrane potential (Fig. 4e). Transmission electron microscopy revealed that LVSPI1 treatment improved the shape of mitochondria, maintained the integrity of mitochondrial morphology, and preserved the double membrane and cristae. However, compared with those in the aging model group, chondrocytes transfected with SiSPI1 exhibited more severe changes in mitochondrial morphology, marked loss of double membranes, disrupted crista structure, and uneven distribution (Fig. 4g). These results suggest that SPI1 can inhibit chondrocyte senescence induced by abnormal mechanical stress.

### SPI1 activates the UPR^mt^ via PERK and inhibits chondrocyte senescence rather than by activating GCN2

**Fig. 5:**
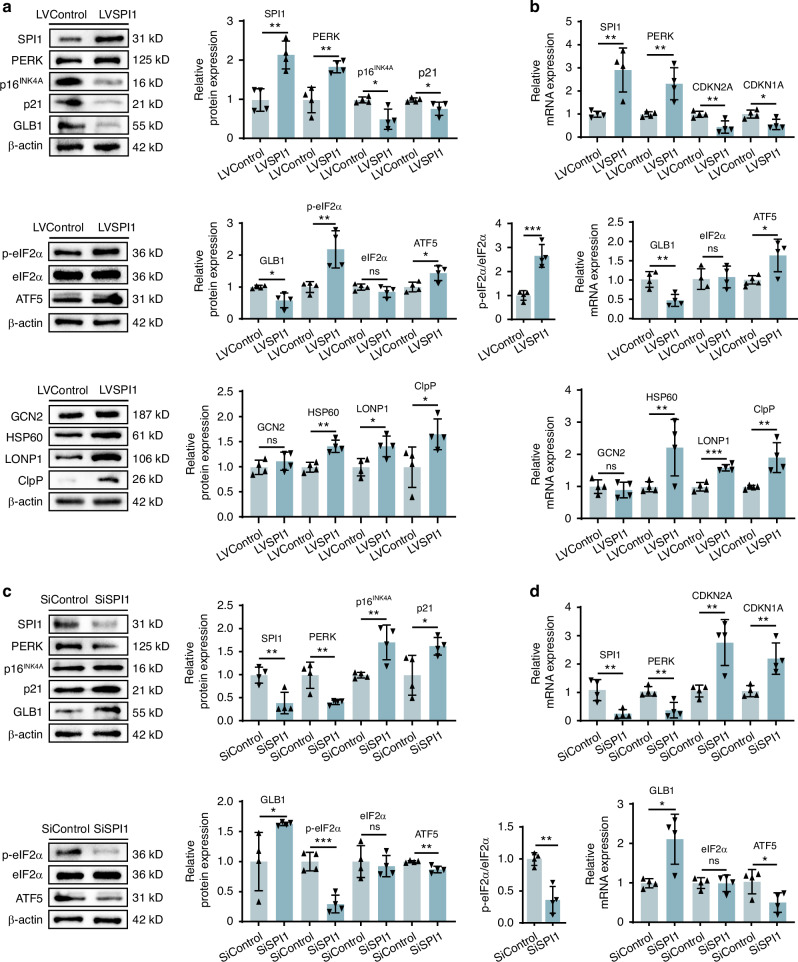
SPI1 activates the UPRmt via PERK and inhibits chondrocyte senescence rather than by activating GCN2. **a** Protein expression of SPI1, PERK, p16^INK4A^, p21, GLB1, GCN2, eIF2α, p-eIF2α, ATF5, HSP60, LONP1 and ClpP was detected by WB in chondrocytes with SPI1 overexpression in model groups. The ratio of p-eIF2α to eIF2α is shown. **b** Gene expression changes of SPI1, PERK, CDKN2A, CDKN1A, GLB1, GCN2, eIF2α, ATF5, HSP60, LONP1 and ClpP was detected by RT-qPCR in chondrocytes with SPI1 overexpression in model groups. **c** Protein expression of SPI1, PERK, p16^INK4A^, p21, GLB1, GCN2, eIF2α, p-eIF2α and ATF5 were detected by WB in chondrocytes with SPI1 inhibition in model groups. The ratio of p-eIF2α to eIF2α is shown. **d** Gene expression changes of SPI1, PERK, CDKN2A, CDKN1A, GLB1, GCN2, eIF2α and ATF5 were detected by RT-qPCR in chondrocytes with SPI1 inhibition in model groups. The cell sample size is *n* = 4. Experiments were independently repeated three times. The data were expressed as mean ± SD. *P < 0.05, **P < 0.01, ***P < 0.001 versus the Control group, ns indicates not significant. *t*-test

Next, we further explored the specific mechanisms by which SPI1 regulates chondrocyte senescence. WB and RT‒qPCR (Fig. 5a, b) revealed that the protein and mRNA levels of PERK were significantly greater in senescent chondrocytes transfected with LVSPI1 than in those in the LVControl group. Interestingly, the level of GCN2 did not change significantly. The protein and mRNA expression levels of CDKN2A, CDKN1A and GLB1 decreased; that is, the degree of chondrocyte senescence decreased. We found that the expression of p-eIF2α and ATF5 increased, and the ratio of p-eIF2α/eIF2α increased, indicating that the overexpression of SPI1 could activate the UPR^mt^. Additionally, HSP60, LONP1, and ClpP protein levels were increased, which is consistent with our previous observation of the mitochondrial status. We then transfected SiSPI1 into senescent chondrocytes, and the protein and mRNA levels of PERK were significantly lower than those in the SiControl group, whereas those of GCN2 remained unchanged. The protein and mRNA levels of CDKN2A, CDKN1A and GLB1 were increased. In addition, the expression levels of p-eIF2α, ATF5, HSP60, LONP1 and ClpP decreased, and the p-eIF2α/eIF2α ratio decreased (Fig. 5c, d and S3a, b). These results suggest that SPI1 activates the UPR^mt^ and inhibits chondrocyte senescence through PERK but not through GCN2.

To further confirm that SPI1 inhibits chondrocyte senescence via PERK-mediated activation of the UPR^mt^, we cotransfected senescent chondrocytes with SiPERK and LVSPI1 and then cotransfected these cells with LVPERK and SiSPI1. In the control group, LVSPI1+SiControl or SiSPI1+LVControl was used to cotransfect senescent chondrocytes. WB and RT‒qPCR results revealed that, compared with those in control cells, PERK knockdown in SPI1-overexpressing senescent chondrocytes increased the protein and mRNA levels of CDKN2A, CDKN1A and GLB1. The levels of p-eIF2α, ATF5, HSP60, LONP1, and ClpP were reduced, and the p-eIF2α/eIF2α ratio was also decreased (Fig. S3c, d). In contrast, overexpression of PERK in a group of senescent chondrocytes in which SPI1 expression was suppressed resulted in decreased levels of CDKN2A, CDKN1A and GLB1. Moreover, the levels of p-eIF2α, ATF5, HSP60, LONP1 and ClpP were increased, and the p-eIF2α/eIF2α ratio was increased (Fig. S3e, f). In SPI1-overexpressing senescent chondrocytes, PERK knockdown exacerbated chondrocyte senescence, increased the expression of IL-6, IL-8, and TNF-α under abnormal mechanical stress, and reduced the function of respiratory chain complexes I-IV. In contrast, overexpression of PERK in SPI1-knockdown senescent chondrocytes alleviated chondrocyte senescence, suppressed the expression of IL-6, IL-8, and TNF-α, and increased the proportion of inhibited complexes I-IV (Fig. S4a-c). PERK knockdown in SPI1-overexpressing senescent chondrocytes significantly reduced the mitochondrial membrane potential. In contrast, overexpression of PERK in senescent chondrocytes with suppressed SPI1 expression restored the mitochondrial membrane potential (Fig. S4d, e). TEM revealed that the morphological changes in mitochondria in the LVSPI1+SiControl group and SiSPI1+LVControl group were mild, and some mitochondrial double membrane and crista structures were disrupted. In the LVSPI1+ SiPERK group, the morphology of the mitochondria was severely damaged, the structure of the double membrane and cristae was unclear, and some mitochondria were even lost and formed vacuolar spaces. In contrast, the morphology of the mitochondria in the SiSPI1+LVPERK group was relatively normal, with a clear and regular arrangement of double membranes and crista structures (Fig. S4f). These results confirmed that SPI1 inhibits chondrocyte senescence via PERK-mediated activation of the UPR^mt^.

### SPI1 binds to the PERK promoter and promotes its transcription

**Fig. 6:**
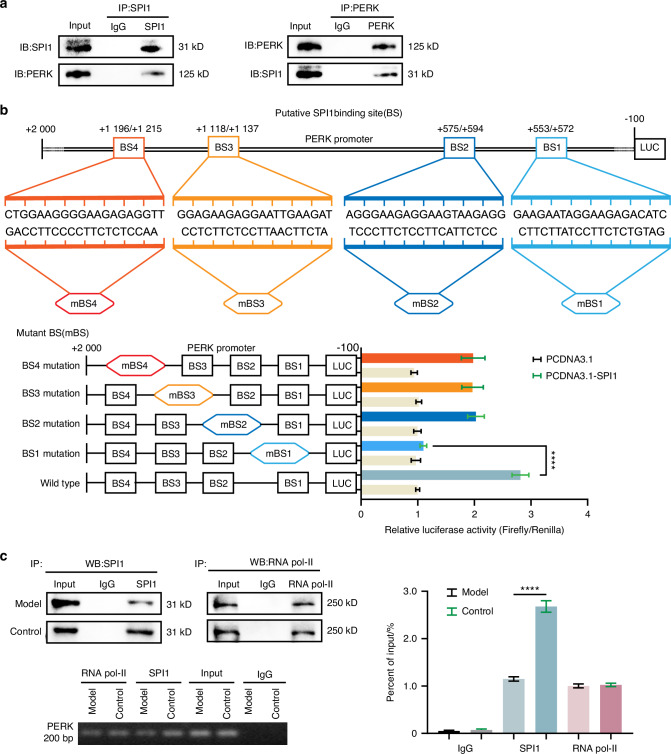
SPI1 binds to the PERK promoter and promotes its transcription. **a** The Co-IP assay was used to detect the interaction of SPI1 and PERK in senescent chondrocytes (*n* = 4). **b** Luciferase reporter assays were used to demonstrate the relationship between SPI1 and PERK promoter in 293T cells. **c** ChIP- RT-qPCR analysis confirmed the immunoprecipitation of SPI1 and BS1 (*n* = 4). Experiments were independently repeated three times. The data were expressed as mean ± SD. ****P < 0.000 1, versus the control group. *t*-test. one-way ANOVA

Given the close association between SPI1 and PERK in chondrocytes, we further investigated their relationship. The results of the co-IP experiments suggested that the SPI1 and PERK proteins may interact in senescent chondrocytes (Fig. 6a). To explore whether SPI1 directly regulates PERK transcription, a dual-luciferase reporter assay was performed, and the results revealed that SPI1 can bind to the PERK promoter and increase its transcription in 293T cells. Furthermore, JASPAR analysis of the PERK promoter sequence (in the range of +2 000-100 relative to the transcription start site of the PERK sequence) revealed four putative SPI1-binding sites (BSs): BS1 (GAAGAATAGGAAGAGACATC; residues +572 to +553), BS2 (AGGGAAGAGGAAGTAAGAGG; residues +594 to +575), BS3 (GGAGAAGAGGAATTGAAGAT; residues +1 137 to +1 118), and BS4 (CTGGAAGGGGAAGAGAGGTT; residues +1 215 to +1 196). Although mutations in BS2, BS3, and BS4 did not significantly affect luciferase activity, mutations in BS1 decreased SPI1-induced luciferase activity (Fig. 6b). ChIP assays revealed a significant decrease in chromosomal DNA containing the PERK promoter, which was immunoprecipitated by the anti-SPI1 antibody, compared with chromosomal DNA containing the PERK promoter, which was immunoprecipitated with the control IgG. ChIP‒RT‒qPCR analysis confirmed the immunoprecipitation of SPI1 and BS1, indicating that the SPI1 protein binds to the PERK promoter and has a significantly stronger effect on senescent chondrocytes than on normal chondrocytes (Fig. 6c). These results suggest that SPI1 directly induces the transcription of PERK in chondrocytes.

### SPI1 and PERK alleviated cartilage degeneration in mechanical overload-induced mice

**Fig. 7:**
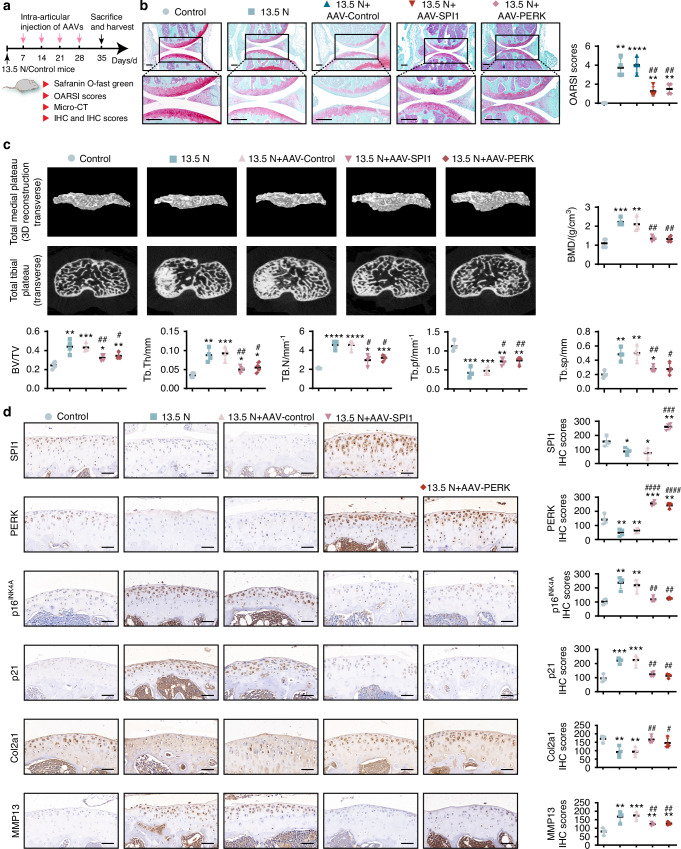
SPI1 and PERK alleviated OA cartilage degeneration in mechanical overload-induced mice. **a** Adeno-associated virus injection into the joints of mice for 4 weeks is schematic, and samples are harvested at 35 days. **b** Safranin O/Fast Green staining and OARSI scores were used to assess cartilage destruction in mouse articular cartilage. Scale bar: 200 μm. **c** Analysis of BMD and knee joint bone histomorphometric parameters using micro-CT data in mice. Original magnification ×50. **d** IHC and IHC scores were used to evaluate the expression of SPI1, PERK, P16 ^INK4A^, P21, Col2a1, and MMP13 after the injection of AAV-SPI1/AAV-PERK. Scale bar: 50 μm. Experiments were independently repeated three times. The animal sample size is *n* = 4. The data were expressed as mean ± SD. *P < 0.05, **P < 0.01, ***P < 0.001, ****P < 0.000 1 versus the Control group, ^#^*P* < 0.05, ^##^*P* < 0.01, ^###^*P* < 0.001, ^####^*P* < 0.000 1 versus the 13.5 N + AAV-Control group. One-way ANOVA

To assess the impact of SPI1 and PERK on the mechanical overload-induced mouse model, the adeno-associated virus vectors AAV-SPI1 and AAV-PERK were injected into mouse knee joints, and AAV-control was used to establish the control group (Fig. 7a). The morphological and histological changes in the model mice were observed via Safranin O/Fast Green staining and micro-CT. Compared with those in the control group, the cartilage degeneration, subchondral sclerosis, and joint space narrowing were significantly alleviated in the AAV-SPI1 and AAV-PERK treatment groups (Fig. 7b, c). IHC revealed a significant increase in SPI1 expression in the 13.5 N + AAV-SPI1 group compared with the control group and a pronounced increase in PERK expression in the 13.5 N + AAV-SPI1 and 13.5 N + AAV-PERK groups compared with the control group. Moreover, the expression of p16^INK4A^, P21 and MMP13 in both the 13.5 N + AAV-SPI1 and the 13.5 N + AAV-PERK groups was markedly lower than that in the control group. The expression of Col2a1 in both the 13.5 N + AAV-SPI1 and 13.5 N + AAV-PERK groups was markedly greater than that in the control group (Fig. 7d). These results confirm the role of SPI1 and PERK in slowing the progression of OA in vivo.

## Discussion

Chondrocyte senescence and the senescence-associated secretory phenotype are the key pathological characteristics of osteoarthritis.^27^ Abnormal mechanical stress significantly contributes to chondrocyte senescence.^28^ Despite the well-established critical role of SPI1 in immune cell-related tumors,^9^ the specific regulatory functions of SPI1 in the aging-related mechanisms underlying OA remain unclear. In this study, we found that SPI1 was significantly downregulated in the cartilage of OA patients and aged mice. Functionally, we found that SPI1 inhibits chondrocyte senescence and the SASP in vitro. SPI1 overexpression significantly inhibited cartilage degeneration, weakening the mechanical overload-induced OA phenotype in vivo. Mechanistically, we revealed that SPI1 reduction promotes OA progression by downregulating PERK gene expression, thus reducing UPR^mt^ signaling.

Aberrant mechanical stress can trigger chondrocyte senescence,^26,29^ and we have successfully established this model. Our findings demonstrate that with increasing duration and intensity of mechanical stress, the expression of SPI1 gradually decreases, whereas the extent of cellular senescence increases. Nevertheless, our findings reveal an intriguing phenomenon in which SPI1 is transiently upregulated during the early stages of mild abnormal stress, followed by an overall downward trajectory. We postulate that this may represent an initial cellular response to mitigate the effects of inevitable aging, with SPI1 maintaining low expression levels under conditions of unavoidable aging. Previous research has focused primarily on the role of SPI1 in blood cells. Philipp et al. reported that restoring SPI1 levels fully restores the long-term regenerative potential of hematopoietic stem cells (HSCs) and controls the transcription of multiple cell cycle regulatory factors to prevent excessive division and depletion of HSCs.^30^ SPI1 has been demonstrated to induce proliferation in erythroid progenitors,^31,32^ but inducible SPI1 overexpression was found to reduce proliferation in HSCs.^33^ Furthermore, extensive discussions on SPI1 have taken place regarding degenerative diseases such as Alzheimer’s disease. Byungwook Kim et al. reported a significant increase in soluble amyloid-β (Aβ) levels, amyloid plaque deposition, and microglial formation upon downregulation of SPI1; conversely, overexpression significantly improved these phenotypes and nutritional deficiency in neuronal processes.^34^ Overexpression also increased resistance to apoptosis in microglia, whereas knockdown increased susceptibility.^35^ These studies indicate that the role of SPI1 in regulating the cell cycle varies across different cell types and diseases. Our findings provide the first evidence that conditional deletion of SPI1 can exacerbate osteoarthritis progression in a mechanical overload-induced mouse model. We have shown that modulating the expression of SPI1 in senescent chondrocytes induced by abnormal mechanical stress can repress the expression of the aging-related genes p16^INK4A^, p21, and GLB1, thereby reducing the SASP and ameliorating cellular aging.

Increasing evidence suggests that the UPR^mt^ plays a crucial role in the aging process and has potential therapeutic implications for age-related diseases. For example, decreased levels of the UPR^mt^ components LONP1 and ClpP have been observed in senescent lung fibroblasts.^36,37^ Downregulation of HSP60 leads to elevated ROS levels and activation of the AMPK‒mTOR pathway, thereby inhibiting glioblastoma cell proliferation.^38^ Our findings revealed lower expression levels of the UPR^mt^-related genes HSP60, LONP1, ClpP, and ATF5 in chondrocytes under abnormal mechanical stress and during aging. The overexpression of SPI1 reversed the downregulation of these genes, inhibited mitochondrial fragmentation, and enhanced oxidative respiratory chain function. These results are consistent with recent research by Zhou Zhibin et al., who demonstrated that nicotinamide riboside protects against OA by enhancing the UPR^mt^ through targeting ATF5.^15^ Our data also revealed decreased PERK expression in OA tissues and cell models, indicating the involvement of PERK in activating the UPR^mt^ in response to abnormal mechanical stress rather than GCN2. Previous studies have reported increases in GRP78 and p-PERK expression with the progression of cartilage degeneration.^39,40^ Additionally, RT‒qPCR array analyses revealed downregulation of PERK expression at both the mRNA and protein levels in OA chondrocytes compared with normal cartilage cells; moreover, overexpression of PERK inhibited type I collagen production while promoting type II collagen production.^41^ Furthermore, disturbances in mitochondrial protein homeostasis lead to activation of the PERK‒eIF2α axis of the UPR, which reduces global protein synthesis and selectively promotes the expression of ATF4, ATF5 and the proapoptotic protein CHOP.^17,42,43^ In our study, we found that the expression of PERK led to the upregulation of UPR^mt^-related genes, thereby inhibiting cell senescence. Furthermore, ATF5 may serve as a sensor for mitochondrial protein dysfunction in chondrocytes. We provide direct evidence that SPI1 binds to PERK and positively regulates its transcription, activating the UPR^mt^ in chondrocytes under abnormal mechanical stress.

In conclusion, our findings indicate that mechanical stress induces chondrocyte senescence and emphasize the protective role of SPI1 in this process through the activation of the UPR^mt^. Targeting the SPI1-mediated activation of the PERK-UPR^mt^ signaling pathway has the potential for combating chondrocyte senescence and treating osteoarthritis.

Our research aimed to investigate the expression of SPI1 in chondrocytes during peak senescence induced by abnormal mechanical stress, as well as the mechanism underlying the potential increase in SPI1 responsiveness during this process, which requires further exploration. PERK is a pivotal regulator of the unfolded protein response and is involved not only in the UPR^mt^ but also in the endoplasmic reticulum unfolded protein response. We did not explore the correlation between PERK and the endoplasmic reticulum unfolded protein response in our model, necessitating further elucidation.

## Materials and Methods

### Ethics statement

This study conformed to the principles of the Declaration of Helsinki and Basel Declaration. All the mice were obtained from the Animal Center of Anhui Medical University. All surgical interventions, treatments, and postoperative animal care procedures were conducted in accordance with the Guide for the Care and Use of Laboratory Animals of the National Institutes of Health. The protocols strictly adhered to the requirements of the Animal Ethics Committee of Anhui Medical University (Ethics Number: LISC 20231477).

### Immunohistochemistry (IHC)

AC samples were acquired from patients who underwent knee arthroplasty at the First Affiliated Hospital of Anhui Medical University (*n* = 10), Normal control cartilage was obtained from hip replacement patients with no prior history of osteoarthritis or rheumatoid arthritis (*n* = 6). 6-μm sections of paraffin-embedded human and mouse tissues were stained with primary antibodies, followed by incubation with secondary antibodies (Santa Cruz, 1:1 000) and a chromogen substrate (Beyotime, Shanghai, China). The staining intensity was semiquantitatively analyzed using the H-score method.^44–46^ Two blinded pathologists evaluated and scored all the sections. Ten fields were observed at 400× magnification. The staining intensity scores ranged from 0 to 3. Total cell counts and numbers of stained cells of each intensity were recorded. H-scores were calculated using a formula. High protein expression was defined as H-scores ≥200. The following antibodies were used: anti-SPI1 (Cell Signaling, 2266S, 1:400), anti-PERK (ab79483; Abcam, IHC 1:100), anti-p16^INK4A^ (ab270058; Abcam, 1:500), anti-p21 (382492, ZENBIO, 1:50), anti-Collagen II (AF0135, Affinity, 1:50), anti-Collagen II (Abcam, ab34712, 1:100), anti-MMP13(Abcam, ab315267, 1:100) and anti-GCN2 (ab134053, Abcam, 1:100).

### Mechanical overload-induced mouse model

Thirty-six male C57BL/6 mice, aged 8 weeks, were utilized to establish the mechanical loading model. Additionally, twelve male C57BL/6 mice aged 20 months served as the natural aging model, while another group of twelve male C57BL/6 mice at 3 months old was designated as the control group. These mice were obtained from Hangzhou Ziyuan Laboratory Animal Technology Co., Ltd. The right knee joints of mice were subjected to axial compressive loads at a frequency of 0.1 Hz, with magnitudes of 4 N, 9 N, or 13.5 N, twice a week for a total of 60 times, using an Electro Force 5100 (USA) device to induce mechanical overload. The control group mice were placed on the test frame, and their knee joints were subjected to a static axial compressive load of 0.5 N for 10 minutes.^47^ After 4 weeks, the mice were euthanized, and knee joint specimens were collected. The samples were then fixed, decalcified, embedded, and sectioned for further analysis.

### Safranin-O/Fast Green staining

Mouse knee joints were harvested, fixed, decalcified, and embedded in paraffin. Sections were stained with Safranin O/Fast Green. Pathological damage was graded according to the OARSI system: stages 0 to 7 represented different degrees of OA activity. Scores were based on affected area percentages: stage 0 (no OA), stage 1 (<10%), stage 2 (10%–25%), stage 3 (25%–50%), and stage 4 (>50%). The total pathology score was the product of grade and stage.^48^

### Quantitative real-time PCR (RT‒qPCR)

Total RNA was extracted from human (OA, *n* = 30; control, *n* = 20) and mouse cartilage tissues or human chondrocytes (1–6 × 10^5^ cells/well) using TRIzol (Takara, Otsu, Japan). The RNA concentration and purity were determined using a NanoDrop spectrophotometer (Thermo Fisher Scientific). cDNA was reverse transcribed with the Prime ScriptTM RT Kit (Takara Bio, Kusatsu, Japan). RT‒qPCR was subsequently used to analyze target gene expression using the SYBR Premix Ex TaqTM II Kit (Accurate Biotechnology, Changsha, China). mRNA expression was analyzed using the 2^-ΔΔCt^ method, with GAPDH serving as an internal reference gene. The primers used by (Sangon, Shanghai, China) are listed in Table S2. Relative gene expression was calculated using Light Cycler 96 software (Roche, Alameda, CA, USA).

### Western blotting (WB)

Total protein was extracted from human and mouse cartilage tissues or human chondrocytes (1–6 × 10^5^ cells/well) using RIPA lysis buffer (Beyotime). Protein concentrations were determined with a BCA assay kit (Beyotime). SDS‒PAGE was used to separate proteins, which were subsequently transferred to PVDF membranes. After blocking, the membranes were incubated with primary antibodies overnight at 4°C. The antibodies used in the study: anti-SPI1 (ab227835, Abcam, 1:1 000; 2266S, Cell Signaling, 1:1 000), anti-PERK (ab229912, ab79483 Abcam, 1:1 000), anti-eIF2α (AF6087, Affinity Biosciences, Changzhou, China, 1:500), Phospho-eIF2 alpha (Ser51)[Ser52] Antibody (AF3087, Affinity, 1:500), anti-p16^INK4A^ (ab270058; Abcam,1:1 000), anti-p21/CDKN1A(382492, ZENBIO, 1:800), anti-β-galactosidase/GLB1 (ab305174, Abcam, 1:1 000; DF3842, Affinity, 1:500), anti-ATF5 (R23549, ZENBIO, 1:500), anti-HSP60 (ab190828, Abcam, 1:100; R24630, ZENBIO, 1:500), anti-ClpP (R389240, ZENBIO, 1:500), anti-LONP1 (161864, ZENBIO, 1:500), anti-GCN2 (ab134053, Abcam, 1:1 000), anti-β-actin (AF7018, Affinity, 1:3 000), and anti-GAPDH (AF7021, Affinity, 1:3 000). Secondary antibodies (mouse or rabbit IgG, 1:10 000) were used to detect the primary antibodies. Chemiluminescent signals were detected using SuperSignal™ West Pico PLUS Chemiluminescent Substrate (Thermo Fisher Scientific). ImageJ software was used for quantitative analysis.

### Chondrocyte isolation and culture of AC samples

AC samples were acquired from patients who underwent knee arthroplasty at the First Affiliated Hospital of Anhui Medical University (*n* = 30). OA samples were chosen on the basis of patient symptoms, which were confirmed by imaging and pathology during surgery. Only OA samples from obese patients with a BMI > 30 [defined by the World Health Organization (WHO) as the weight in kilograms divided by the square of height in meters (kg/m^2^)] were included. Normal control cartilage was obtained from hip replacement patients with no prior history of osteoarthritis or rheumatoid arthritis (*n* = 20). Patient consent and procedures were approved by the Ethics Committee of the First Affiliated Hospital of Anhui Medical University (Approval Number: 2023-583). The basic patient information is shown in Table S1. Cartilage samples were washed, cut into 1 × 1 × 1 mm chips, trypsinized, and digested with type II collagenase (Solarbio, Beijing, China). Chondrocytes were collected, cultured in DMEM/F12 (HyClone, Logan, UT, United States) supplemented with 10% FBS (HyClone), and passaged when they reached 80%–90% confluence. Early passages were used to maintain the phenotype.

### Immunofluorescence (IF)

For IF of human chondrocyte slides, the cells were fixed, permeabilized, and blocked. Chondrocytes were incubated with antibodies overnight at 4 °C, followed by incubation with secondary antibodies (Santa Cruz, CA, USA; 1:1 000) and DAPI (Beyotime) staining. The slides were observed under a fluorescence microscope (Olympus IX 81). The following antibodies were used: anti-SPI1 (2266S, Cell Signaling, IF 1:100), anti-PERK (ab65142; Abcam, 1:500).

### Establishment of a model of abnormal mechanical stress-induced chondrocyte senescence in human OA samples

Primary OA chondrocytes (1–6 × 10^5^ cells/well), following digestion with trypsin, were seeded on the surface membranes of a Bio Flexcell six-well plate, and 2.5 mL of complete culture medium was added. The cells were then subjected to periodic mechanical tensile stress using an FX-4000T in vitro cell mechanical loading system in a CO_2_ incubator at 37 °C. The time gradient for mechanical stress was set to 0 h, 12 h, 24 h, 48 h, and 60 h. The intensity of the mechanical stress was set to 0%, 5%, 10%, 15%, 20%, and 25%. In subsequent experiments, OA chondrocytes were subjected to mechanical tensile stress for either 0 h (control group) or 48 h. Each well of the membrane six-well plate was subjected to a deformation magnitude of 20%, with a deformation frequency of 6 cycles/min.^44,47^

### Cell viability assessment

The viability of senescent chondrocytes (1–6 × 10^3^ cells/well) was evaluated using a Cell Counting Kit-8 (CCK-8) (Beyotime). After induction by mechanical stress or viral infection, all the groups were seeded in a single 96-well plate (5 × 10^3^ cells/well). Following washing with PBS, 100 μL of fresh culture medium containing 10% CCK-8 reagent was added to each well, and the plate was then incubated at 37 °C for 2 h. The absorbance at 450 nm was measured using a microplate reader (Leica Microsystems, Wetzlar, Germany).

### Senescence-associated β-galactosidase staining

The senescence status of primary chondrocytes was assessed using a cellular senescence staining kit (Beyotime). The proportion of senescence-associated β-galactosidase (SA-β-Gal)-positive cells was calculated by analyzing five randomly selected low-power fields.

### ELISA

The concentrations of IL-6 (#EH2IL6, Invitrogen, Carlsbad, CA, USA), IL-8 (#88-8086-88, Invitrogen, Carlsbad, CA, USA), and TNF-α (#BMS223-4, Invitrogen, Carlsbad, CA, USA) in the cell supernatants were quantified using enzyme-linked immunosorbent assay (ELISA) kits. The assays were conducted following the protocols provided by the manufacturer.

### Mitochondrial Membrane Potential Assessment

Fluorescence detection: Senescent chondrocytes (1–6 × 10^5^ cells/well) in six-well plates were treated, washed with PBS, and incubated with JC-1 (Servicebio, Wuhan, China) working solution (5 L JC-1 200x in 1 mL of buffer) for 25 mins at 37 °C. After washing and adding fresh medium, the cells were observed under a fluorescence microscope (DM6B; Leica, Germany). For flow cytometry detection, the cells were trypsinized, mixed with JC-1 working solution, and incubated. After washing and resuspension, flow cytometry (BD Celesta) was used for analysis.

### Mitochondrial respiratory complex activity measurement

The activities of NADH ubiquinone reductase (complex I), succinate ubiquinone reductase (complex II), ubiquinol cytochrome C reductase (complex III), and cytochrome C oxidase (complex IV) were measured in primary mouse chondrocytes. These enzyme activities were determined using assay kits (A089-1, A089-2, A089-3, A089-4) from Nanjing Jiancheng Bioengineering Institute, China, according to the manufacturer’s instructions.

### Transmission electron microscopy for assessment of mitochondrial morphology

Senescent chondrocytes (1–6 × 10^5^ cells/well) were fixed in 2.5% glutaraldehyde solution at 4 °C, dehydrated, embedded in EPON812, and sectioned into ultrathin sections (50–70 nm). These sections were stained and observed under a transmission electron microscope (Talos L120C G2; Thermo Fisher Scientific, Waltham, MA, United States).

### Establishment of a mechanical overload-induced mouse model in SPI1 CKO mice

Mice bearing SPI1^fl/fl^ (C57BL/6 J mice were obtained from Anhui Medical University) with loxP sites flanking exons 5 and 6 of the SPI1 gene were crossed with a transgenic mouse line expressing Cre recombinase under the control of the type II collagen promoter and inducible by tamoxifen administration (Col2a1-CreER^T2^), resulting in SPI1 CKO mice.^31^ Eight-week-old SPI1 CKO male mice and their respective control group SPI1^+/+^ littermates were intraperitoneally injected with 100 μg/g body weight tamoxifen (TM, Sigma‒Aldrich) daily for 5 days. A mechanical load of 13.5 N was then applied to wild-type and SPI1-CKO mice, and also mechanical load of 0.5 N for 10 mins was used as a control. There were 4 mice in each group. Samples were collected after 4 weeks for further analysis.^47^

### Micro-CT assessment

To assess bone damage, the right knee joints of the mice sacrificed at 4 weeks were fixed in a 4% paraformaldehyde solution (Beyotime) and scanned for 160 minutes using an ex vivo micro-CT scanner (Skyscan1174 X-ray Micro-CT, Bruker) with settings of 50 kV and 800 μA, achieving a resolution of 14.5 μm. The data were then reconstructed using N-Reconn and CTvox software to create 3D images of the joints and to measure bone mineral density (BMD) and other morphometric indicators. Bone destruction in the right knee joint was evaluated, with the BMD serving as a comparative measure of bone damage. The region of interest (ROI) within the right knee was analyzed for the following morphometric properties: (1) bone volume to total tissue volume ratio (BV/TV), (2) trabecular number (Tb.N), (3) trabecular thickness (Tb.Th), (4) trabecular separation (Tb.Sp), and (5) trabecular pattern factor (Tb.Pf).

### Virus transduction

Senescent chondrocytes were plated in Flexcell six-well plates at approximately 60% confluence (1–6 × 10^5^ cells/well) with 1 mL of fresh medium. Polybrene (10 µL) was added to aid in transduction. LVSPI1 or LVPERK lentivirus was added to each well. After 6 h, the medium was adjusted to 2 mL and gently shaken. After 24 h, the medium was replaced with fresh medium. Forty-eight h later, GFP expression in chondrocytes was assessed under a fluorescence microscope (Olympus IX 81) to evaluate transduction efficiency.

### SiRNA transfection

SPI1 and PERK were silenced using SiSPI1 and SiPERK, respectively, which were obtained from RiboBio (Guangzhou, China), along with their negative controls. Chondrocytes were transfected with these SiRNAs at a concentration of 50 nmol/L using the riboFECT™ CP Transfection Kit (RiboBio) following the manufacturer’s protocol. Transfection efficiency was assessed 48–72 h posttransfection via RT‒qPCR and WB analysis. The SiSPI1 sequence was GCAAGAAGATGACCA, the SiPERK sequence was 5’-GAAGCUACAUUGUCUAUUU-3’, and the control sequence was 5’-AACCACUCAACUUUUUCCCAA-3’.

### Coimmunoprecipitation (Co-IP) assay

Senescent chondrocytes (1–6 × 10^5^ cells/well) were lysed in RIPA buffer (Beyotime) at 4 °C for 30 mins. After centrifugation, the supernatants were incubated overnight at 4 °C with anti-SPI1 (Abcam, ab302623, 1:30; Cell Signaling, 2266S, 1:25), anti-PERK (Immunoway, YM8183, 1:50) and normal IgG (2729S, Cell Signaling) antibody-bound protein A/G magnetic beads (78610, Thermo Fisher Scientific). Following centrifugation, the bead complexes were washed, boiled in 10% SDS, and subjected to WB analysis.

### Dual-luciferase reporter assay (Dual-LUC)

The 293 cells used in this study were cultured in Dulbecco’s modified Eagle’s medium (DMEM, Gibco, USA) supplemented with 10% fetal bovine serum (HyClone) and 1% penicillin/streptomycin (Beyotime). To confirm the ability of SPI1 to bind to the PERK promoter and promote its transcription, we constructed various plasmids: pcDNA3.1, pcDNA-SPI1, PGL3-Basic, PGL3-PERK-WT, PGL3-PERK-Mut1, PGL3-PERK-Mut2, PGL3-PERK-Mut3, PGL3-PERK-Mut4, and pRL-TK. These plasmids were cotransfected into 293T cells seeded in 24-well plates at 50%–60% confluence (1–2 × 10^5^ cells/well). Transfection was performed using LipofectamineTM 3000 (solution B). After 48 h of culture, luciferase activity was measured using a Dual-Luciferase Reporter Gene Assay System Kit (Beyotime). Firefly luciferase activity was normalized to Renilla luciferase activity.

### Bioinformatics software prediction

The JASPAR database was used to predict potential binding sites of SPI1 in the PERK promoter, and subsequent experimental validation was subsequently performed.

### Chromatin immunoprecipitation (ChIP) assay

In the ChIP assay, senescent chondrocytes were crosslinked, sonicated, and incubated with a specific SPI1 antibody overnight at 4 °C. The antibody‒protein‒DNA complexes were eluted, and the interaction between SPI1 and the PERK promoter was assessed using RT‒qPCR. Antibody: anti-SPI1 (Abcam, ab302623, 1:30; Cell Signaling, 2266S, 1:25), anti-RNA polymerase II antibody (13523, Cell Signaling) and normal IgG (2729S, Cell Signaling) antibody-bound protein A/G magnetic beads (78610, Thermo Fisher Scientific). The sequences of the primers used for the ChIP-RT‒qPCR assay targeting the PERK promoter were as follows: 5’- GTTAGAAGAATAGGAAGAGACATC-3’ (sense) and 5’- CTCTGATGTCTCTTCCTATTCTTC-3’ (antisense).

### Animal model treatment

Twenty 8-week-old male wild-type C57BL/6J mice were obtained from Anhui Medical University’s Animal Center. The mice were housed under standard conditions with a 12-h light/dark cycle and had free access to water and standard food. The mice were randomly divided into 5 groups (6 mice/group): Group 1, normal control (G1); Group 2, 13.5 N mechanical overload-induced model mice (G2); Group 3, 13.5 N mechanical overload-induced mice injected intra-articularly with AAV-control for 4 weeks (G3); Group 4, 13.5 N mechanical overload-induced mice injected intra-articularly with AAV-SPI1 for 4 weeks (G4); and Group 5, 13.5 N mechanical overload-induced mice injected intra-articularly with AAV-PERK for 4 weeks (G5). The mice were sacrificed for sampling on day 35. The injected adenoviruses had a concentration of 1 × 10^12^ vg/mL, and the volume injected was 10 μL. Adenovirus transfection typically results in gene expression within 1–2 days, and this gene expression lasts for 7–14 days.^49^

### Statistical analysis

Differences between two groups were assessed using an unpaired, two-tailed *t*-test. Multiple comparisons were conducted using one-way analysis of variance (ANOVA) followed by an LSD post hoc test. All the experiments were conducted in triplicate. Statistical significance was set at a two-tailed *P* value < 0.05. IBM SPSS Statistics 29 and GraphPad Prism (v. 8.0.1) were used for the data analysis.

## Supplementary information

Supplementary information

Supporting data values

Unedited blot and gel images

## Data Availability

All data included in this study are available upon reasonable request of corresponding authors.
