# Supplementary material for: SPI1 activates mitochondrial unfolded response signaling to inhibit chondrocyte senescence and relieves osteoarthritis
Source: Bone Res. 2025 Apr 14;13:47. doi: 10.1038/s41413-025-00421-4 (PMC11997156; doi:10.1038/s41413-025-00421-4)
Supplement: Supplementary file 1 — Supplementary information [file 41413_2025_421_MOESM1_ESM.docx]

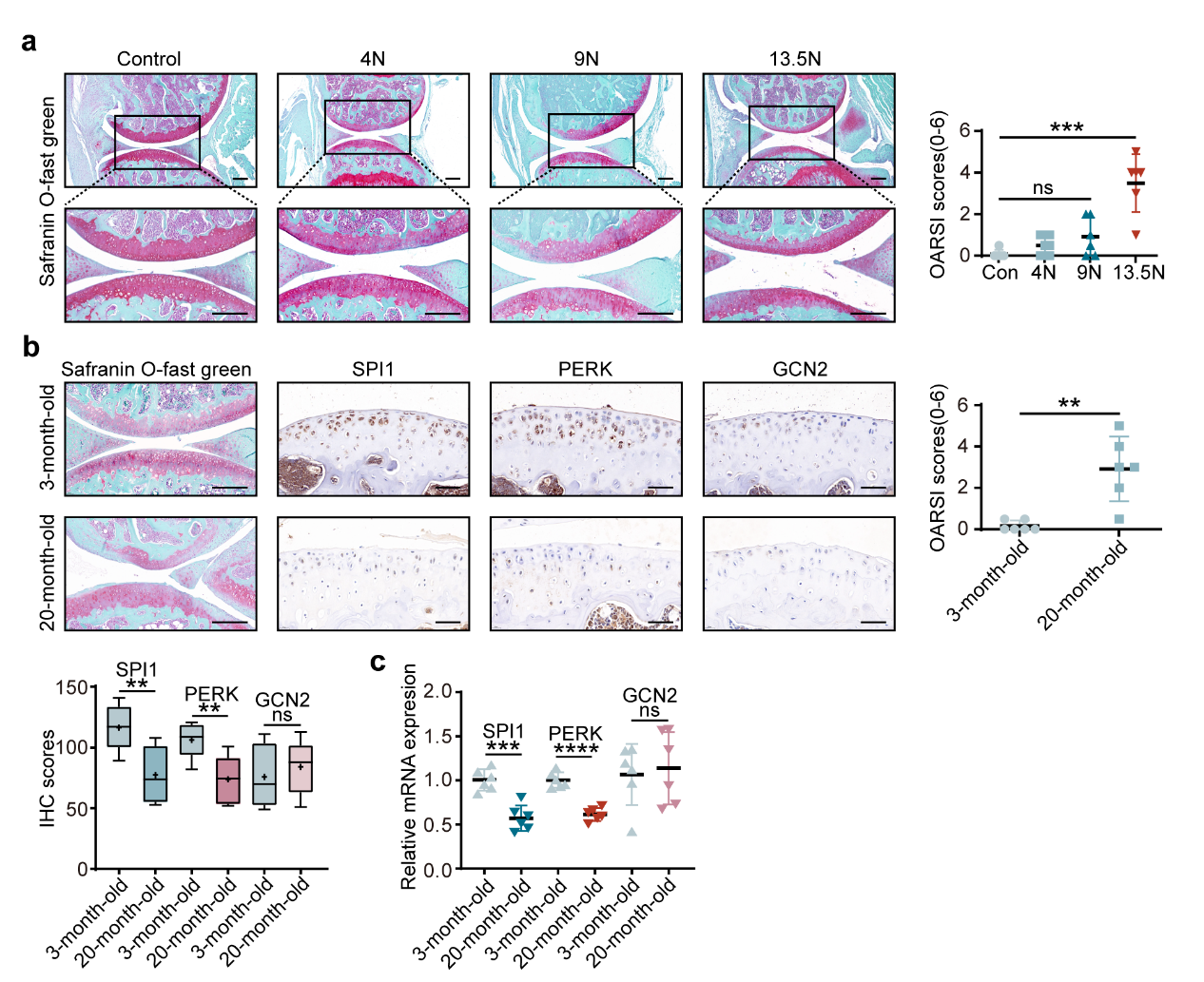


**Fig. S1 SPI1 and PERK expression was significantly decreased in osteoarthritic articular cartilage. a** Safranin O/Fast Green staining and OARSI scoring were used to evaluate cartilage damage in the control group as well as in 4N, 9N, and 13.5N mechanical overload-induced model mice. Scale bars: 200 μm. **b** Safranin O/Fast Green staining and OARSI scoring were used to evaluate cartilage damage in young mice (3-mon-old) as well as in aged mice (20-mon-old mice). Scale bars: 200 μm. IHC and IHC scores were used to evaluate the expression of SPI1, PERK and GCN2 in the cartilage tissues of aged mice and young mice. Scale bar: 50 μm. **c** RT-qPCR was employed to assess the mRNA expression of SPI1, PERK and GCN2 in the cartilage tissues of aged mice and young mice. The sample size is n=6. Experiments were independently repeated three times. The data were expressed as mean ± SD. ns, no statistical significance. **p < 0.01, ***p < 0.001, ****p < 0.0001 versus the control group, ns indicates not significant. t-test.

**
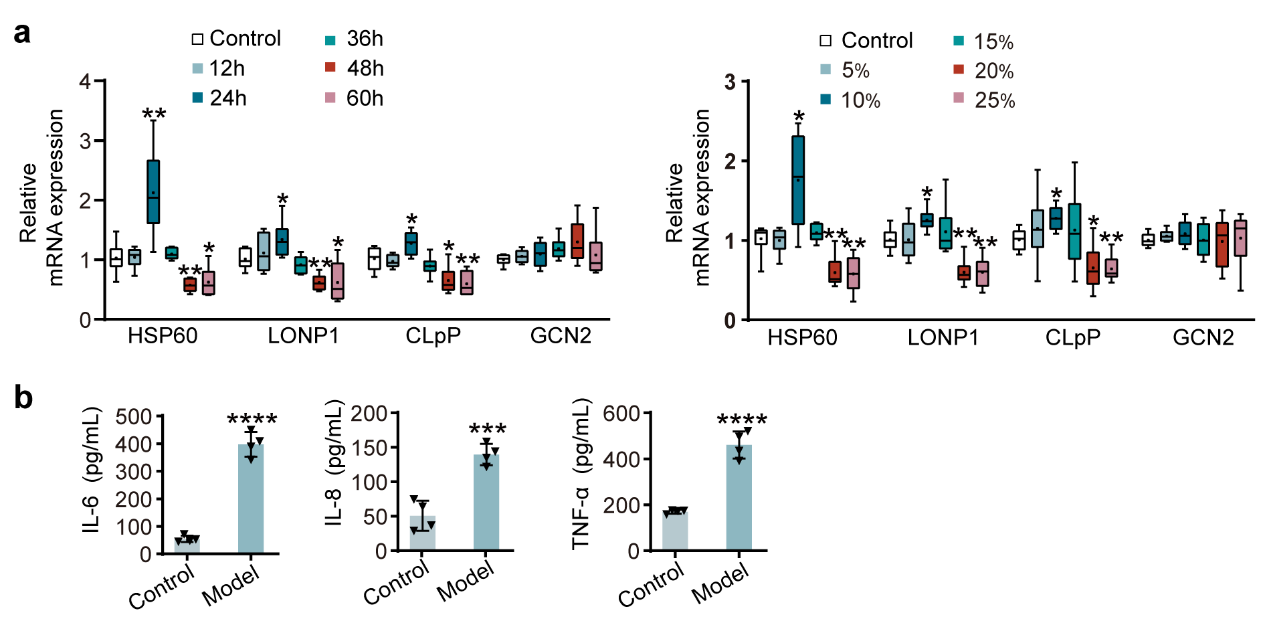
**

**Fig. S2** **The expression of GCN2, HSP60, LONP1 and ClpP in the Chondrocytes under mechanical stress time and stress gradient. a** RT-qPCR was employed to assess the mRNA expression of GCN2, HSP60, LONP1 and ClpP in the Chondrocytes under different mechanical stress times and stress gradients (n=6 per group). **b** IL-6, IL-8 and TNF-α in the culture supernatant of model chondrocytes were measured by ELISA (n=4 per group). Experiments were independently repeated three times. Data are expressed as mean ± SD. *p<0.05, **p<0.01, ***p<0.001, ****p<0.0001 versus the control group. t-test.


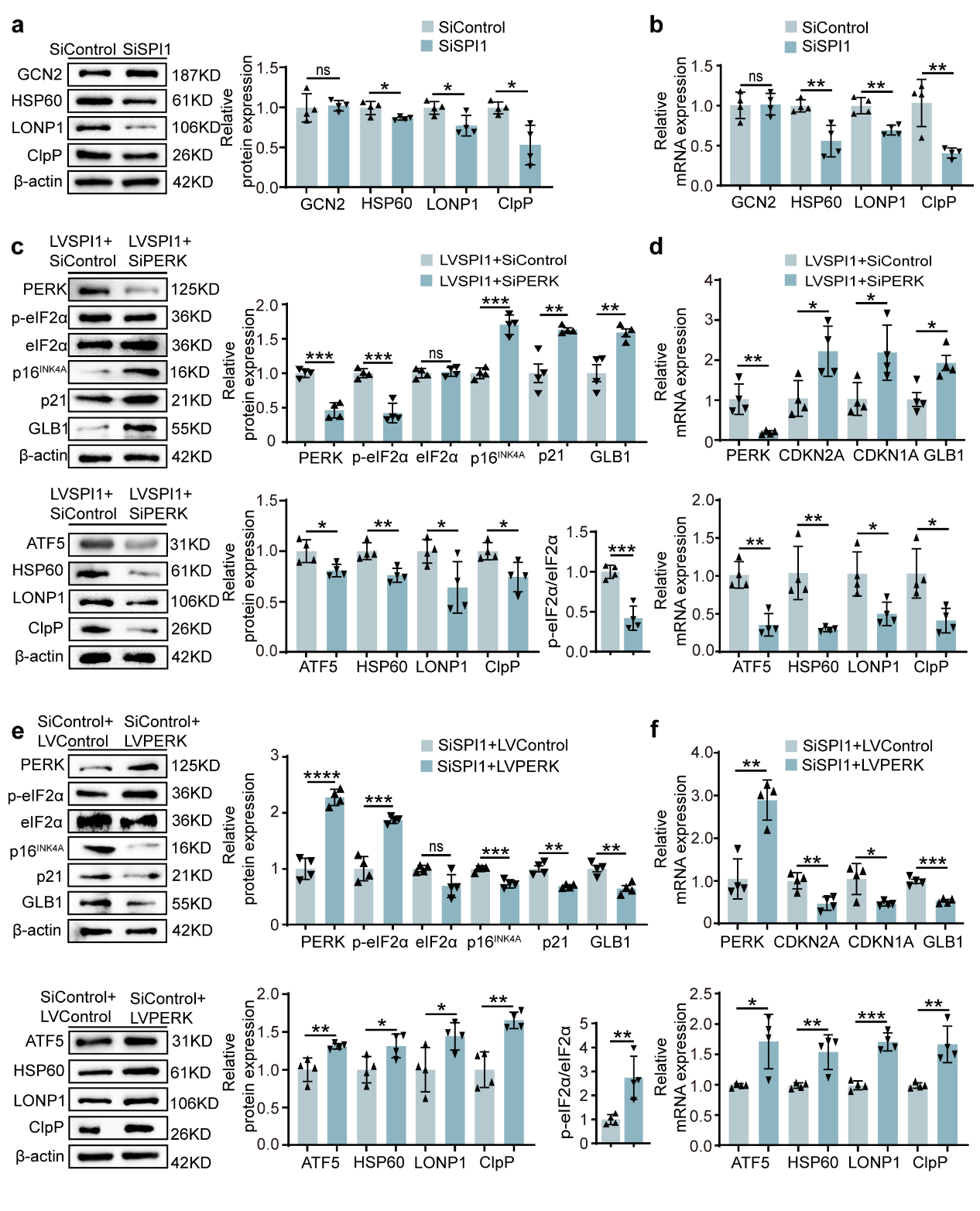


**Fig. S3 SPI1 activates the UPR^mt^ via PERK and inhibits chondrocyte senescence rather than by activating GCN2. a** Protein expression of GCN2, HSP60, LONP1, and ClpP was assessed by WB in chondrocytes with the inhibition of SPI1 in model groups. **b** Gene expression changes of HSP60, LONP1, and ClpP were determined by RT-qPCR in chondrocytes with the inhibition of SPI1 in model groups. **c** Protein expression of SPI1, PERK, p16^INK4A^, p21, GLB1, p-eIF2α, eIF2α, ATF5, HSP60, LONP1, and ClpP was examined by WB in chondrocytes from model groups with SPI1 overexpression and PERK inhibition. The ratio of p-eIF2α to eIF2α is shown. **d** Gene expression of SPI1, PERK, CDKN2A, CDKN1A, GLB1, ATF5, HSP60, LONP1, and ClpP was analyzed by RT-qPCR in chondrocytes from model groups with SPI1 overexpression and PERK inhibition. **e** Protein expression of PERK, p16^INK4A^, p21, GLB1, p-eIF2α, eIF2α, ATF5, HSP60, LONP1, and ClpP was assessed by WB in chondrocytes from model groups with SPI1 inhibition and PERK overexpression. The ratio of p-eIF2α to eIF2α is shown. **f** Gene expression of PERK, p16^INK4A^, p21, GLB1, ATF5, HSP60, LONP1, and ClpP was determined by RT-qPCR in chondrocytes from model groups with SPI1 inhibition and PERK overexpression. The cell sample size is n=4. Experiments were independently repeated three times. The data were expressed as mean ± SD. *p<0.05, **p<0.01, ***p<0.001, ****p<0.0001 versus the Control group. ns indicates not significant. t-test.


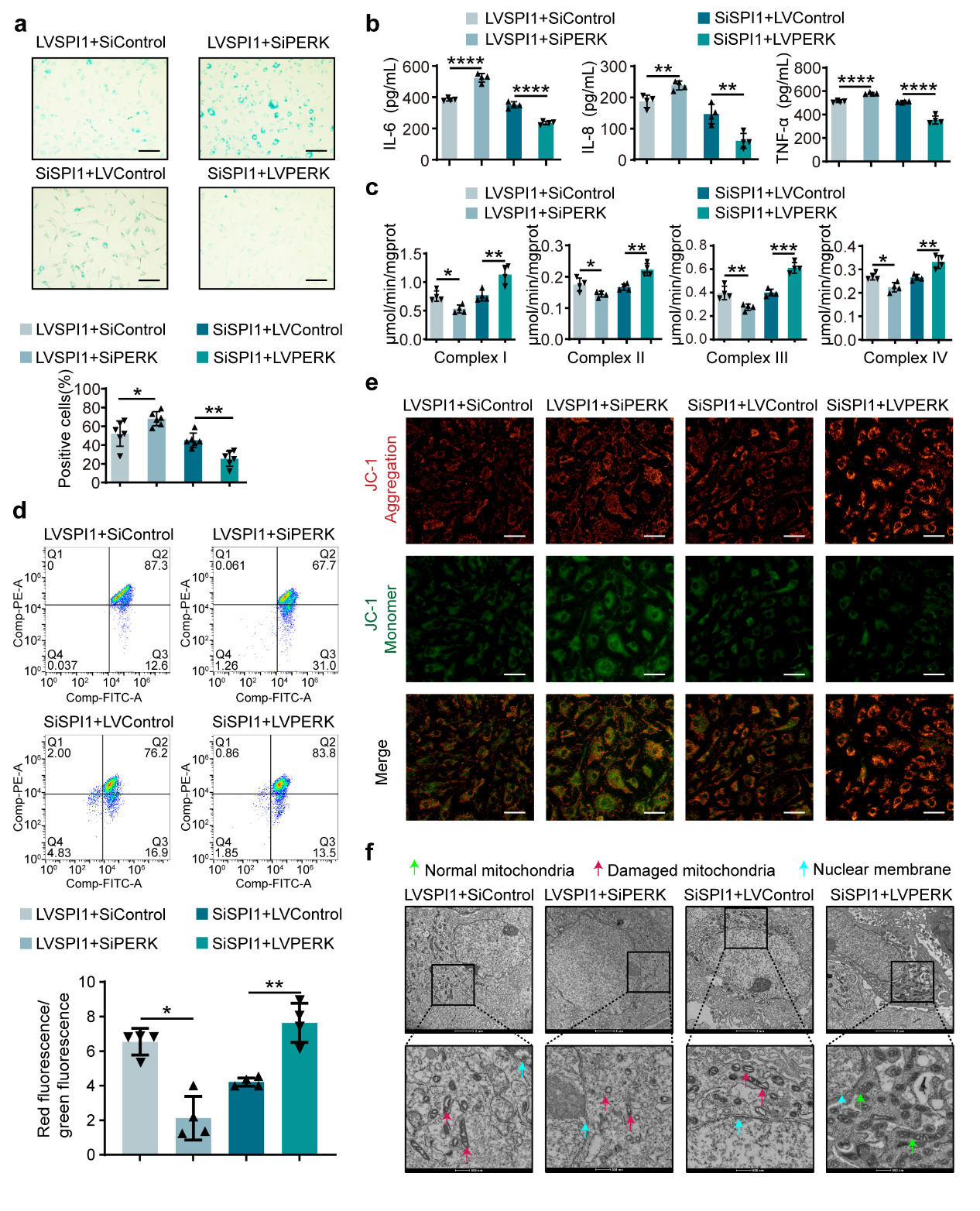


**Fig. S4 SPI1 activates the UPR^mt^ via PERK and inhibits chondrocyte senescence rather than by activating GCN2. a** The proportion of senescent chondrocytes with overexpression or inhibition of SPI1 or PERK was determined by β-galactosidase staining. (n=6 per group). Scale bars: 50 μm. **b** IL-6, IL-8, and TNF-α in the culture supernatant of chondrocytes from model groups with overexpression or inhibition of SPI1 or PERK were measured by ELISA. **c** Mitochondrial respiratory chain complexes I-IV were determined in chondrocytes from model groups with overexpression or inhibition of SPI1 or PERK. **d-e** Flow cytometry analysis (JC-1) and microscopic observation were used to evaluate the mitochondrial membrane potential after inhibition or overexpression of SPI1 or PERK in the chondrocytes from model groups. Scale bars: 50 μm **f** TEM was employed to observe the morphology and structure of mitochondria after the inhibition or overexpression of SPI1 or PERK in chondrocytes from model groups. Scale bars: 2 μm and 500 nm. The sample size is n=4. Experiments were independently repeated three times. The data were expressed as mean ± SD. *p<0.05, **p<0.01, ***p<0.001, ****p<0.0001 versus the Control group. t-test.

**Table**

| **Table S1.** Baseline patient characteristics (n=50) | | | |
| --- | --- | --- | --- |
| Patients | Knee osteoarthritis (n=30) | Femoral neck fracture (n=20) |  |
| Age in years, mean (SD) | 66 (6.9) | 64 (4.7) |  |
| Female, n (%) | 21(70) | 10(50) |  |
| Duration of  osteoarthritis (year), mean (SD) | 6.7(4.9) |  |  |
| HAQ, mean (SD) | 1.8(0.7) |  |  |
| NSAID, n (%) | 24 (80) |  |  |
| Previous biological therapy, n (%)  HSS score, mean (SD)  HKA Angle, mean (SD) | 3(10).  47.5(7.21)  171.41(5.44) |  |  |

HAQ, health assessment questionnaire; NSAID, non-steroidal anti-inflammatory drug;

HSS score, hospital for special surgery knee score; HKA Angle, hip-knee-ankle angle

**Table S2.** Primer sequences used in the study.

| Primer name | Primer sequences |
| --- | --- |
| H-SPI1F | GTGCCCTATGACACGGATCTA |
| H-SPI1R | AGTCCCAGTAATGGTCGCTAT |
| H-PERKF | ACGATGAGACAGAGTTGCGAC |
| H-PERKR | ATCCAAGGCAGCAATTCTCCC |
| H-CDKN2AF | GATCCAGGTGGGTAGAAGGTC |
| H-CDKN2AR | CCCCTGCAAACTTCGTCCT |
| H-CDKN1AF | TGTCCGTCAGAACCCATGC |
| H-CDKN1AR | AAAGTCGAAGTTCCATCGCTC |
| H-GLB1F | TATACTGGCTGGCTAGATCACTG |
| H-GLB1R | GGCAAAATTGGTCCCACCTATAA |
| H-ATF5F | TGGCTCGTAGACTATGGGAAA |
| H-ATF5R | ATCAACTCGCTCAGTCATCCA |
| H-EIF2αF | CCGCTCTTGACAGTCCGAG |
| H-EIF2αR | GCAGTAGTCCCTTGTTAGTGACA |
| H-GCN2F | AAATGCCCACCTACCTATCCA |
| H-GCN2R | CCTCCCCACAGTGTTTCTTGG |
| H-HSP60F | ATGCTTCGGTTACCCACAGTC |
| H-HSP60R | AGCCCGAGTGAGATGAGGAG |
| H-LONP1F | GACGATCCCCGATGTGTTTCC |
| H-LONP1R | GGGCGAGACGAACTTTCCTT |
| H-CLPPF | TTGCCAGCCTTGTTATCGCA |
| H-CLPPR | GGTTGAGGATGTACTGCATCG |
| H-GAPDHF | CAGGAGGCATTGCTGATGAT |
| H-GAPDHR | GAAGGCTGGGGCTCATTT |
| M-SPI1F | TTACAGGCGTGCAAAATGGAA |
| M-SPI1R | GACGTTGGTATAGCTCTGAATCG |
| M-PERKF | CGCGTCGGAGACAGTGTTT |
| M-PERKR | GTCCTCCACGGTCACTTCG |
| M-CDKN2AF | CGCAGGTTCTTGGTCACTGT |
| M-CDKN2AR | TGTTCACGAAAGCCAGAGCG |
| M-CDKN1AF | CCTGGTGATGTCCGACCTG |
| M-CDKN1AR | CCATGAGCGCATCGCAATC |
| M-GLB1F | GCACGGCATCTATAATGTCACC |
| M-GLB1R | GTATCGGAATGGCTGTCCATC |
| M-ATF5F | TGGGCTGGCTCGTAGACTAT |
| M-ATF5R | GTCATCCAATCAGAGAAGCCG |
| M-EIF2αF | CACGGTGCTTCCCAGAGAATC |
| M-EIF2αR | GTCCCTTGTTAGCGACATTGA |
| M-GCN2F | CCCGGACATACTCCTCAGGAA |
| M-GCN2R | GGCTACCCACAGAGAAATGGA |
| M-HSP60F | ACAGTCCTTCGCCAGATGAGA |
| M-HSP60R | ACCTTGAAGCATTAAGGCTCG |
| M-LONP1F | ATGACCGTCCCGGATGTGT |
| M-LONP1R | CCTCCACGATCTTGATAAAGCG |
| M-CLPPF | GCCTTGCCGTGCATTTCTC |
| M-CLPPR | CTGCTCCACCACTATGGGGA |
| M-GAPDHF | GGTTGTCTCCTGCGACTTCA |
| M-GAPDHR | TGGTCCAGGGTTTCTTACTCC |

**H**, primers of human species; **M**, primers of mouse species.
