# Supplementary material for: SPI1 activates mitochondrial unfolded response signaling to inhibit chondrocyte senescence and relieves osteoarthritis
Source: Bone Res. 2025 Apr 14;13:47. doi: 10.1038/s41413-025-00421-4 (PMC11997156; doi:10.1038/s41413-025-00421-4)

**Figure 1**
**Human cartilage tissue**

Some gel images in the same section share the same β-actin gel image.

SPI1 31KD

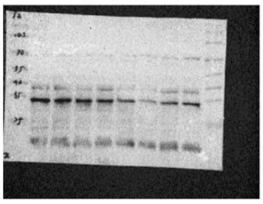

β-actin 42KD

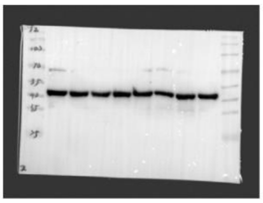

PEKR 125KD

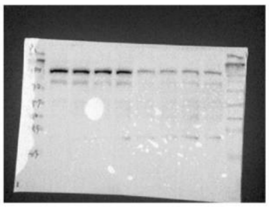

β-actin 42KD

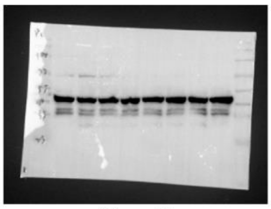

GCN2 187KD

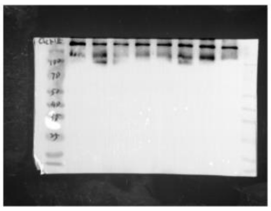

β-actin 42KD

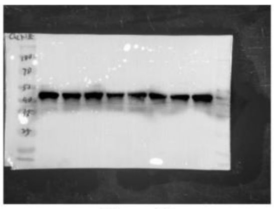

**Human chondrocytes**

SPI1 31KD

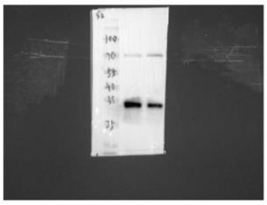

β-actin 42KD

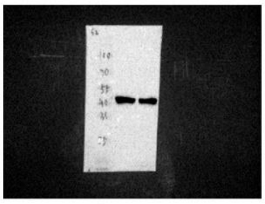

PEKR 125KD

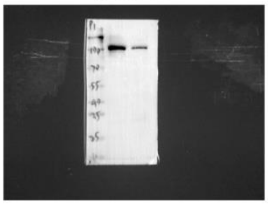

β-actin 42KD

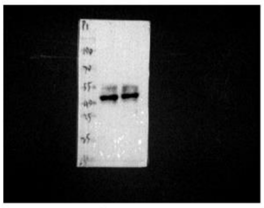

GCN2 187KD

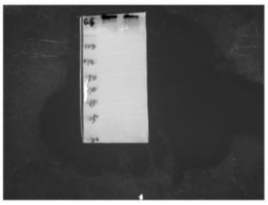

β-actin 42KD

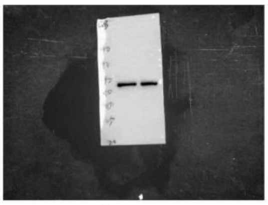

## Figure 2

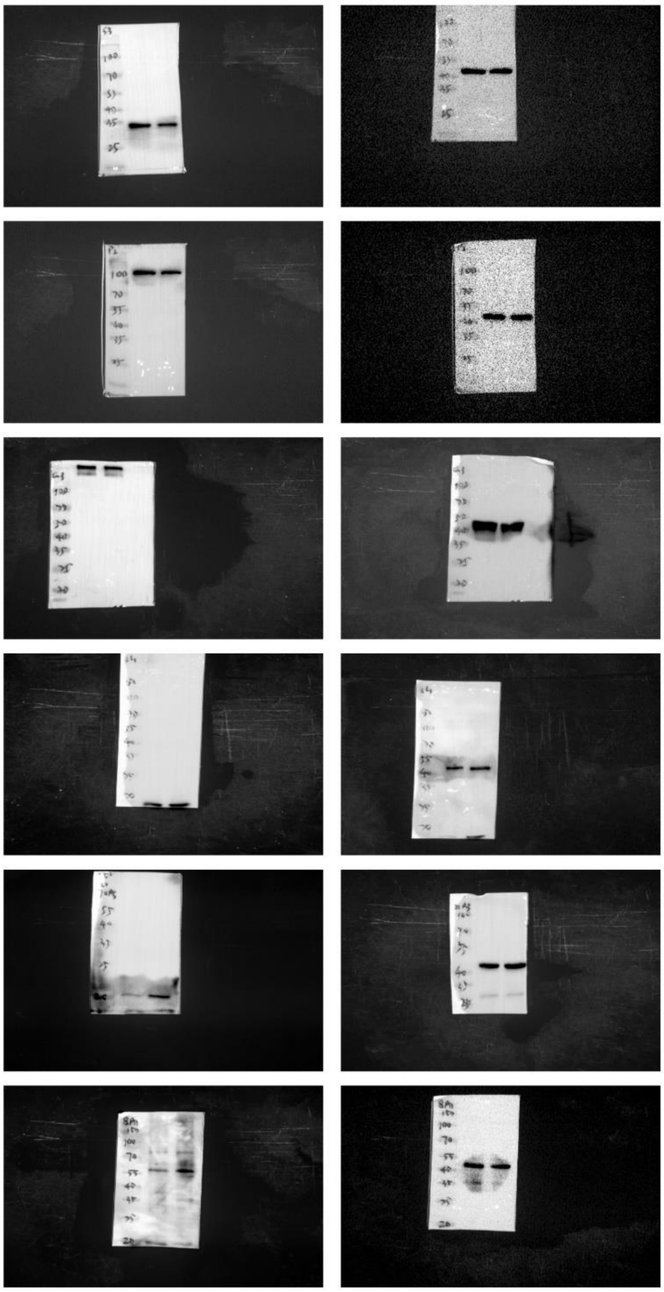

SPI1 31KD — β-actin 42KD

PEKR 125KD — β-actin 42KD

GCN2 187KD — β-actin 42KD

p16$^{INKA4}$ 16KD — β-actin 42KD

p21 21KD — β-actin 42KD

GLB1 55KD — β-actin 42KD

**Figure 5 ab① LV-SPI1**

SPI1
31KD

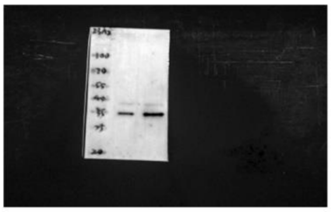

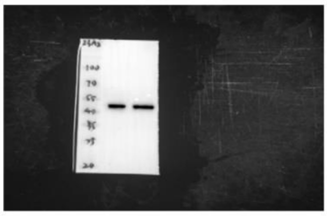

β-actin
42KD

PEKR
125KD

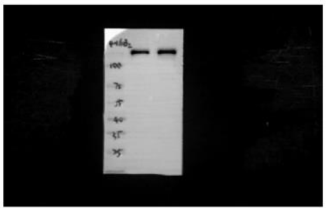

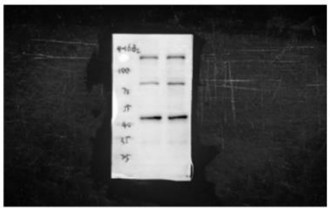

β-actin
42KD

$p16^{INKA4}$
16KD

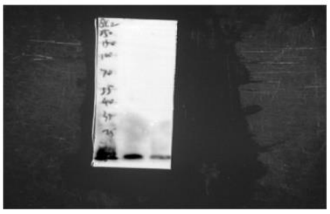

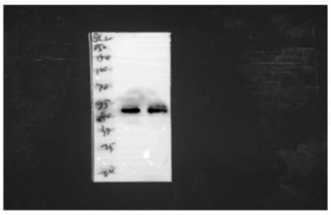

β-actin
42KD

p21
21KD

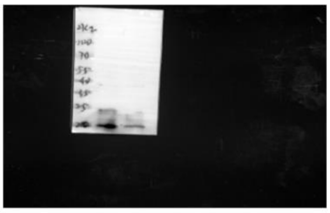

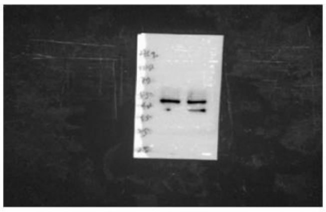

β-actin
42KD

GLB1
55KD

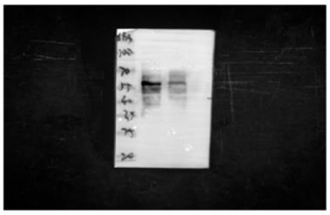

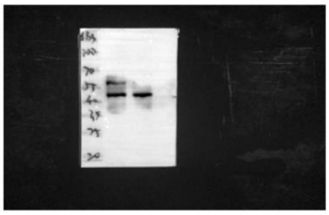

β-actin
42KD

**Figure 5ab** ③ LVSPI1

**LONP1**
**106KD**

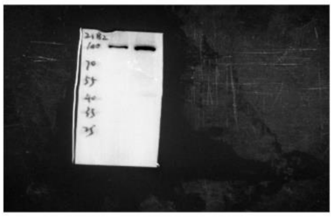

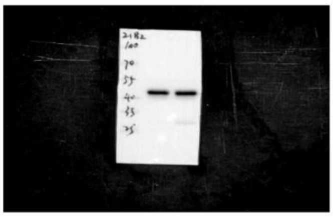

**β-actin**
**42KD**

**CLPP**
**26KD**

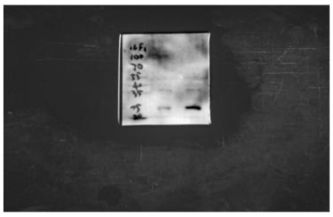

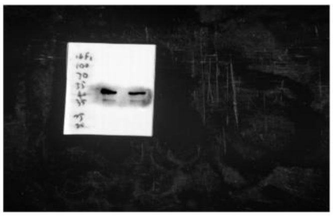

**β-actin**
**42KD**

**Figure 5 cd** ① **siSPI1**

**SPI1**
**31KD**

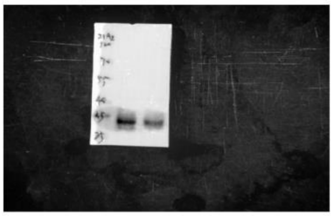

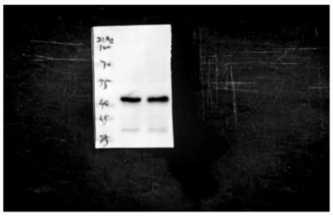

**β-actin**
**42KD**

**PEKR**
**125KD**

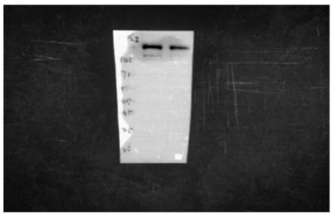

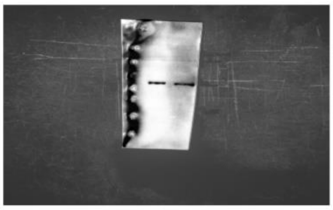

**β-actin**
**42KD**

**$p16^{INKA4}$**
**16KD**

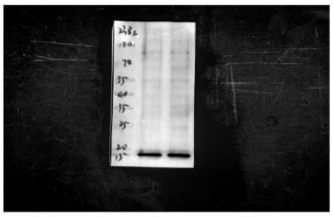

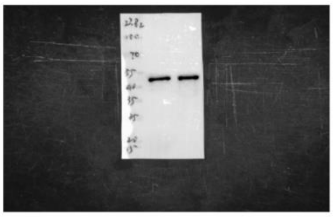

**β-actin**
**42KD**

**Figure 5ab** ② LVSPI1

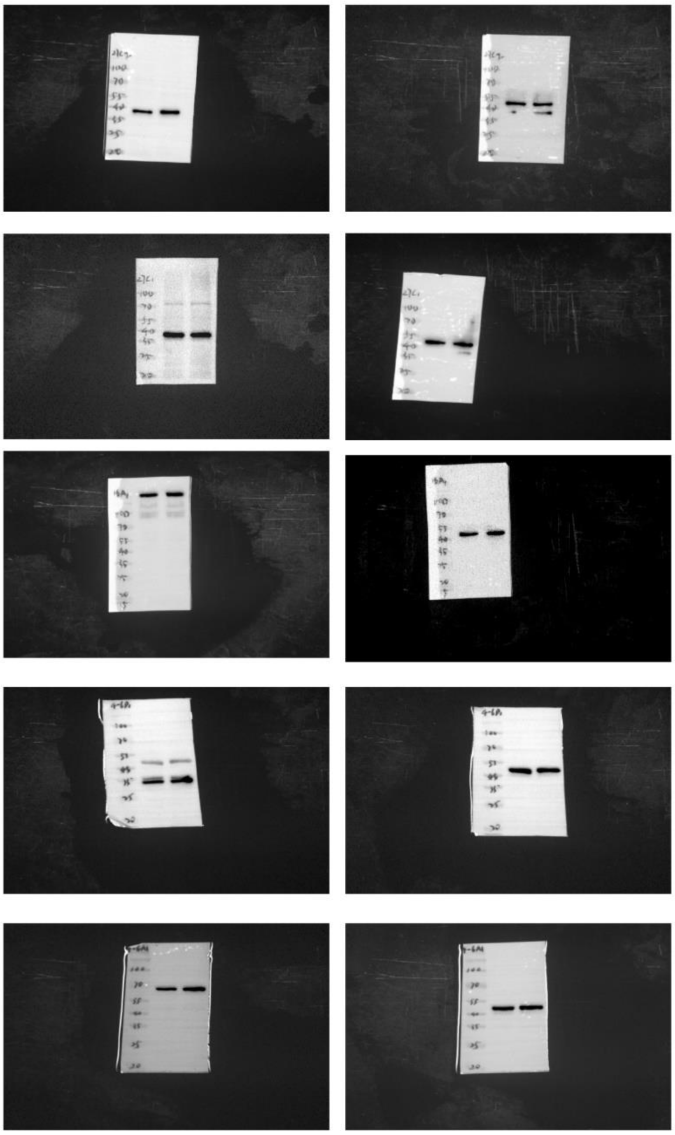

P-EIF2α 36KD — β-actin 42KD

same β-actin gel image as that of p21 in Figure 5 ab.

EIF2α 36KD — β-actin 42KD

GCN2 187KD — β-actin 42KD

ATF5 31KD — β-actin 42KD

HSP60 60KD — β-actin 42KD

**Figure 5cd** ② SI-SPI1

p21
21KD

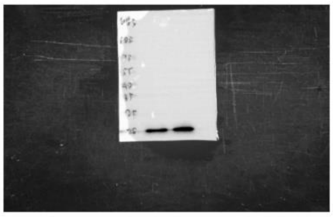

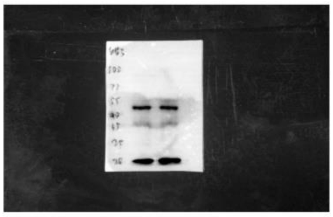

β-actin
42KD

GLB1
55KD

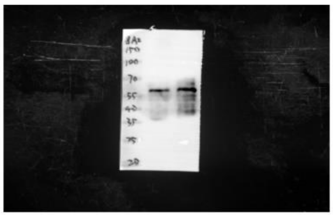

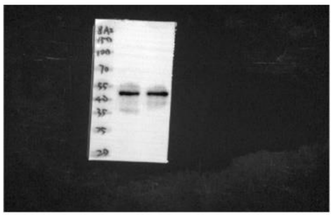

β-actin
42KD

GCN2
187KD

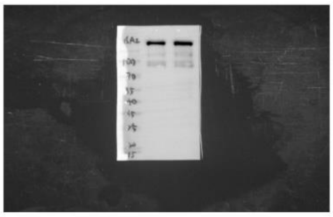

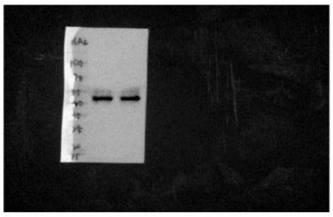

β-actin
42KD

P-EIF2α
36KD

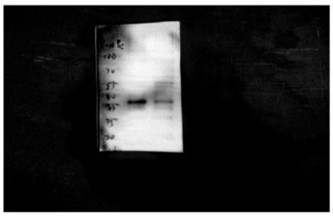

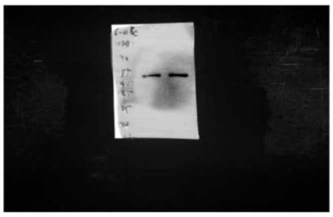

β-actin
42KD

EIF2α
36KD

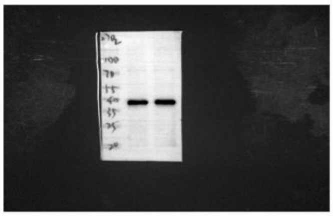

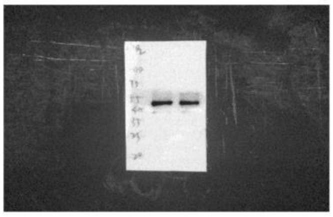

β-actin
42KD

**Figure S3ab** SI-SPI1

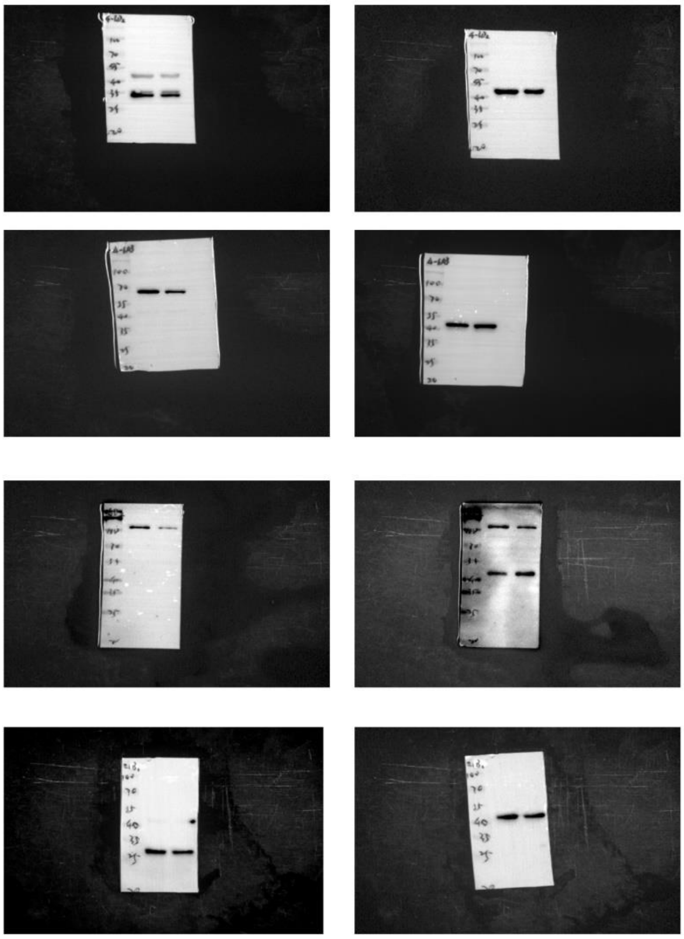

ATF5
31KD

β-actin
42KD

HSP60
60KD

β-actin
42KD

LONP1
106KD

β-actin
42KD

CLPP
26KD

β-actin
42KD

**Figure S3 cd siPERK-①**

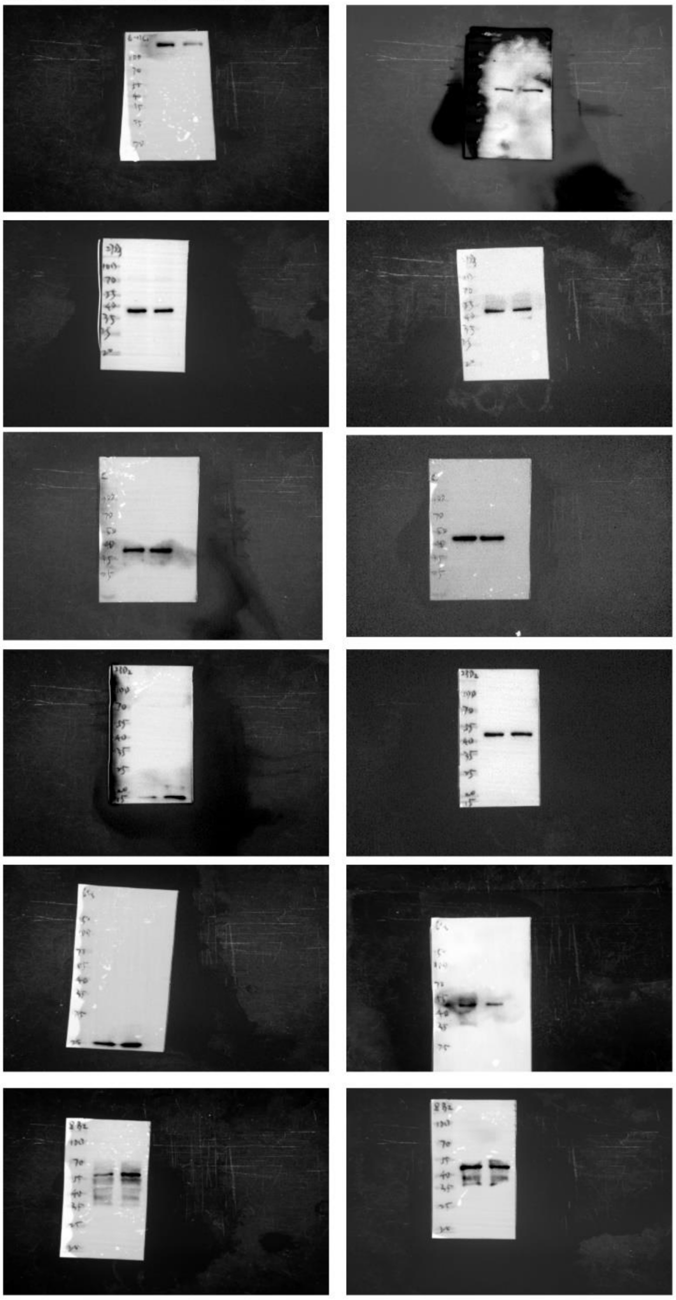

PEKR 125KD | β-actin 42KD

P-EIF2α 36KD | β-actin 42KD

EIF2α 36KD | β-actin 42KD

p16$^{INKA4}$ 16KD | β-actin 42KD

p21 21KD | β-actin 42KD

GLB1 55KD | β-actin 42KD

Figure S3 cd **si**PERK

ATF5
31KD

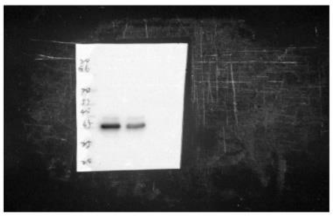

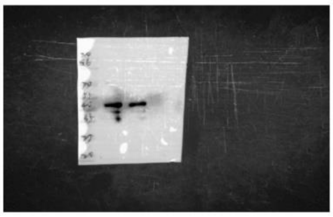

β-actin
42KD

HSP60
60KD

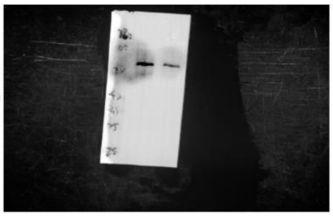

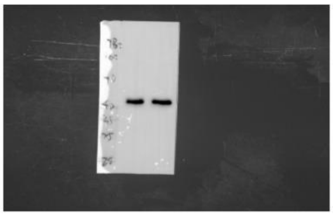

β-actin
42KD

LONP1
106KD

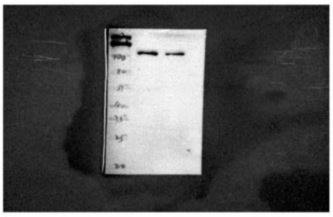

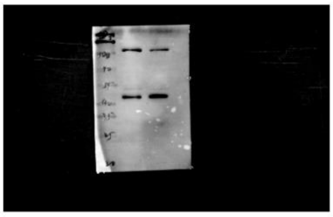

β-actin
42KD

CLPP
26KD

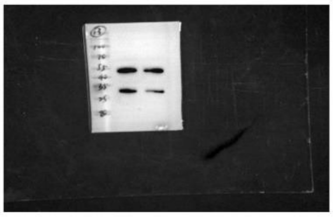

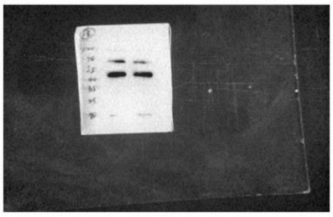

β-actin
42KD

Figure S3 ef ① siPERK

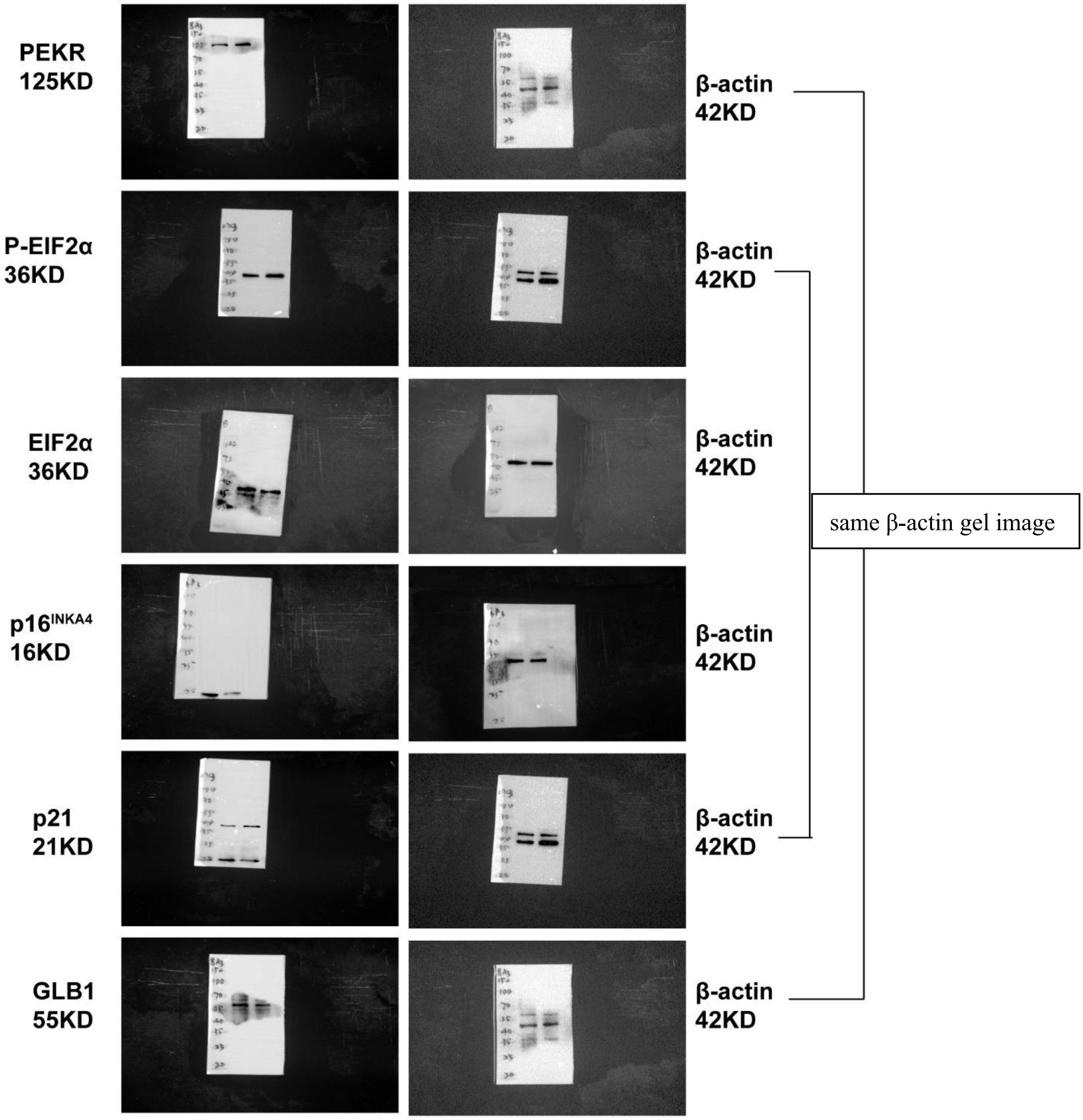

**Figure S3 ef LV-PERK**

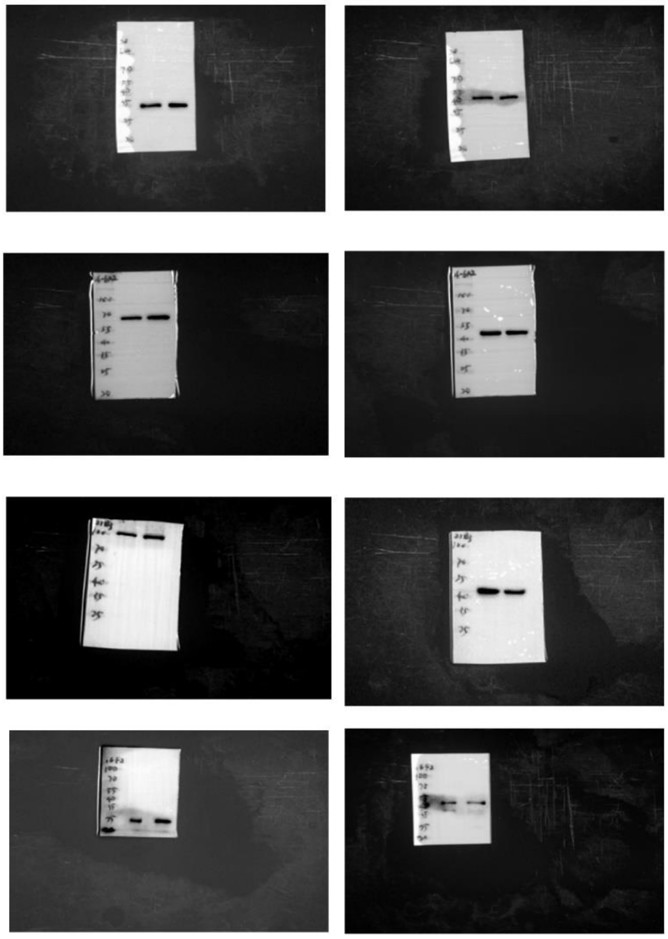

ATF5
31KD

β-actin
42KD

HSP60
60KD

β-actin
42KD

LONP1
106KD

β-actin
42KD

CLPP
26KD

β-actin
42KD

Figure 6, CO-IP

IP:SPI1

SPI1
31KD

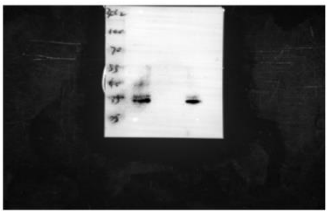

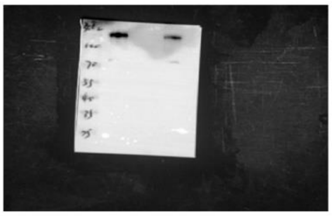

PEKR
125KD

IP:PERK

SPI1
31KD

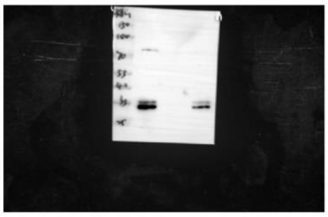

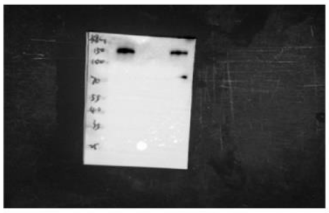

PEKR
125KD

Model

SPI1
31KD

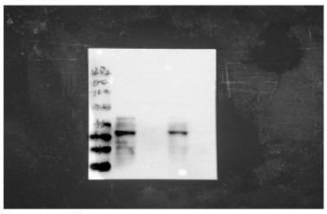

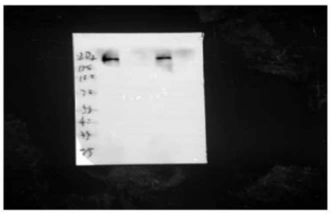

RNA pol II
250KD

Conrol

SPI1
31KD

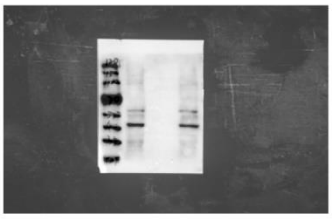

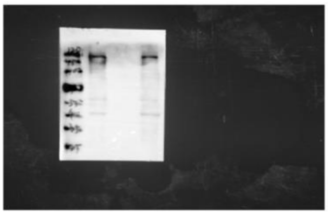

RNA pol II
250KD

200bp→

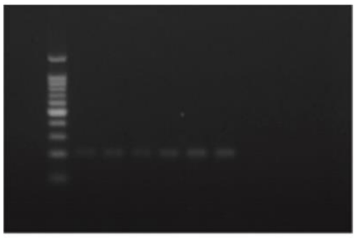

Supplement: Supplementary file 3 — Unedited blot and gel images [file 41413_2025_421_MOESM3_ESM.pdf]
